# Olanzapine suppresses mPFC activity-norepinephrine releasing to alleviate CLOCK-enhanced cancer stemness under chronic stress

**DOI:** 10.1186/s12964-024-01747-y

**Published:** 2024-07-25

**Authors:** Jinxin Lu, Xiaoyu Zhang, Keyu Su, Huandong Luo, Congcong Liu, Yuqing Yang, Bin He, Cenxin Wang, Zhuoran Zhao, Xianxian Liu, Xu Wang, Peixuan Meng, Dekang Lv, Chunli Wang, Keith W. Kelley, Ling Wang, Bai Cui, Quentin Liu, Fei Peng

**Affiliations:** 1 Institute of Cancer Stem Cell, Dalian Medical University, Dalian, China; 2 State Key Laboratory of Oncology in South China, Cancer Center, Sun Yat-sen University, Guangzhou, China; 3 Department of Oncology, the First Affiliated Hospital of Dalian Medical University, Dalian, China; 4 Department of Pathology, College of Medicine, Department of Animal Sciences, College of ACES, University of Illinois at Urbana-Champaign, Urbana, IL USA

**Keywords:** Olanzapine, Chronic stress, Norepinephrine, CLOCK transcription, Gemcitabine resistance, Cancer stemness, mPFC activity

## Abstract

**Background:**

Olanzapine (OLZ) reverses chronic stress-induced anxiety. Chronic stress promotes cancer development via abnormal neuro-endocrine activation. However, how intervention of brain-body interaction reverses chronic stress-induced tumorigenesis remains elusive.

**Methods:**

*Kras*^*LSL−G12D/WT*^ lung cancer model and LLC1 syngeneic tumor model were used to study the effect of OLZ on cancer stemness and anxiety-like behaviors. Cancer stemness was evaluated by qPCR, western-blotting, immunohistology staining and flow-cytometry analysis of stemness markers, and cancer stem-like function was assessed by serial dilution tumorigenesis in mice and extreme limiting dilution analysis in primary tumor cells. Anxiety-like behaviors in mice were detected by elevated plus maze and open field test. Depression-like behaviors in mice were detected by tail suspension test. Anxiety and depression states in human were assessed by Hospital Anxiety and Depression Scale (HADS). Chemo-sensitivity of lung cancer was assessed by in vivo syngeneic tumor model and in vitro CCK-8 assay in lung cancer cell lines.

**Results:**

In this study, we found that OLZ reversed chronic stress-enhanced lung tumorigenesis in both *Kras*^*LSL−G12D/WT*^ lung cancer model and LLC1 syngeneic tumor model. OLZ relieved anxiety and depression-like behaviors by suppressing neuro-activity in the mPFC and reducing norepinephrine (NE) releasing under chronic stress. NE activated ADRB2-cAMP-PKA-CREB pathway to promote CLOCK transcription, leading to cancer stem-like traits. As such, CLOCK-deficiency or OLZ reverses NE/chronic stress-induced gemcitabine (GEM) resistance in lung cancer. Of note, tumoral CLOCK expression is positively associated with stress status, serum NE level and poor prognosis in lung cancer patients.

**Conclusion:**

We identify a new mechanism by which OLZ ameliorates chronic stress-enhanced tumorigenesis and chemoresistance. OLZ suppresses mPFC-NE-CLOCK axis to reverse chronic stress-induced anxiety-like behaviors and lung cancer stemness. Decreased NE-releasing prevents activation of ADRB2-cAMP-PKA-CREB pathway to inhibit CLOCK transcription, thus reversing lung cancer stem-like traits and chemoresistance under chronic stress.

**Supplementary Information:**

The online version contains supplementary material available at 10.1186/s12964-024-01747-y.

## Introduction

As an atypical antipsychotic agent for schizophrenia and bipolar I disorder [1], olanzapine (OLZ) reverses chronic stress-induced anxiety by remodeling neuropeptides (BDNF), neuroactive hormones (ACTH, dopamine and allopregnanolone) [2–4] and neural activities [5]. Cancer is one of the life-threatening diseases closely associated with chronic stress [6]. Chronic stress abnormally activates hypothalamic-pituitary-adrenal (HPA) axis and sympathetic nervous system (SNS) to facilitate various malignant features of cancer, including tumor growth, cancer stemness and chemoresistance [6]. Specific brain regions are actively involved in chronic stress-enhanced tumorigenesis. Optogenetic activation of the ventral tegmental area (VTA^TH^) projections in the medial prefrontal cortex (mPFC) reduces the level of NE and corticosterone in serum and reverses stress-induced breast cancer progression, suggesting VTA^TH^-mPFC as an essential neural circuit in chronic stress-enhanced tumorigenesis [7]. Meanwhile, OLZ potentially modulates the function of mPFC, as OLZ increases the release of dopamine and ACh in mPFC [8, 9]. However, whether OLZ stimulates specific brain regions to control neuro-endocrine activity that modulates chronic stress-induced anxiety and tumorigenesis remains unclear.

NE is one of the critical stress-related neurotransmitters that activates adrenergic receptors, leading to Gαs-mediated activation of adenylyl cyclase, which converts adenosine triphosphate (ATP) into cyclic 3′-5′ adenosine monophosphate (cAMP). The increased cAMP further activates protein kinase A (PKA) to phosphorylate transcription factor cAMP response element binding protein (CREB) responsible for transactivation of its target genes [10]. The cAMP-PKA pathway is activated under chronic stress to promote tumor initiation [11] and angiogenesis [12]. Moreover, NE and epinephrine-induced SNS activation controls rhythmic expression of core clock genes in murine liver, indicating a potential role of chronic stress in modulating peripheral clock [13]. The circadian regulator CLOCK-BMAL1 complex maintains cancer stem cells and promotes immunosuppression in glioblastoma [14]. Yet, whether chronic stress-induced NE modulates circadian clock to promote cancer stemness remains elusive.

Lung cancer is the major cause of cancer-related death globally [15]. Clinical relapse of lung cancer is largely attributed to chemoresistance to first-line anti-cancer agents [16], such as carboplatin (CBP), cisplatin (CDDP), gemcitabine (GEM), pemetrexed (PEM) and paclitaxel (PTX) [17]. In recent years, immunotherapy targeting immune checkpoint and other anti-cancer therapy targeting cancer metabolism and circadian clock have emerged to improve chemosensitivity [16, 18]. OLZ has been accepted as a component of antiemetic regimens for patients receiving chemotherapy for cancer [19]. Meanwhile, OLZ is proved to sensitize cancer cell to chemotherapeutic agents and radiotherapy in vitro [20, 21]. Nevertheless, the mechanism by which OLZ improves chemotherapy in chronic stress-enhanced lung cancer is poorly understood. Thus, investigating the therapeutic role of OLZ in chronic stress-related cancer will benefit patients with cancer and chronic stress undergoing chemoresistance.

In our study, we found that OLZ suppressed chronic stress-enhanced lung tumorigenesis and anxiety-like behaviors by inhibiting stress-induced mPFC activation and NE releasing. Mechanistically, NE activated ADRB2-cAMP-PKA-CREB pathway to promote CLOCK transcription that sustains lung cancer stem-like traits. Thus, OLZ suppresses mPFC-NE-CLOCK axis to alleviate chronic stress-enhanced cancer stemness. Importantly, OLZ sensitized lung cancer cells to chemotherapy with GEM under chronic stress.

## Methods

### Mice

The wild-type (WT) C57BL/6J mice were purchased from GemPharmatech. *Kras*^*LSL−G12D/WT*^ mice (Strain No. NM-KI-190003) on C57BL/6J background were obtained from the Shanghai Model Organisms Center. All animals were housed at 24 ± 2 °C and 50% ± 10% relative humidity and subjected to a strict 24 h light-dark cycle (08:00 light on, 20:00 light off). Food and water are provided at will. At the beginning of the experiment, the age, weight and sex of the mice were randomly assigned among the groups without significant differences. At the end of the experiment, animals were euthanized by inhaling isoflurane, followed by cervical dislocation, and tissue was collected for subsequent experiments. All animal studies were conducted in accordance with the Animal Care Guidelines of Dalian Medical University and approved by the Institutional Animal Care and Use Committee (IACUC) of Dalian Medical University (AEE18018).

### Cell culture

The human embryonic renal epithelial cell HEK 293T, mouse lung cancer cell LLC1, and human lung cancer unit NCI-H1299 were purchased from the American Center for the Collection of Typical Cultures (ATCC). HEK 293T and LLC1 cells were cultured in DMEM medium (Gibco, C11995500BT) supplemented with 10% fetal bovine serum (FBS, Gibco, 10270-106). NCI-H1299 cells were cultured in RPMI-1640 medium (Gibco, C11875500BT) containing 10% FBS. All cell lines were cultured in the standard medium recommended by ATCC and authenticated before purchase by standard short tandem repeat DNA-typing methodology. Maintain all cells in a moist atmosphere of 5% CO_2_ at 37 ℃ with 0.1% penicillin- streptomycin (Beyotime, C0222) in all cell culture system. Petri dishes and cell culture plates were purchased from Jet Bio-Filtration Co., Ltd (Guangzhou, China) and NEST Biotechnology Co. Ltd. (Wuxi, China).

### Lung cancer mouse models

#### *Kras*^*LSL−G12D/WT*^ lung cancer model

Induction of lung tumors in *Kras*^*LSL−G12D/WT*^ mice by intratracheal delivery of genetically engineered adenovirus. Male and female *Kras*^*LSL−G12D/WT*^ mice with 4-week were stunned with isoflurane inhalation in a biosecurity cabinet. Head and limbs were fixed, mouse trachea exposed and adenovirus expressing GFP-Cre-recombinase (AAV-GFP-Cre, 30 µL) was injected (Hanbio, LN 06032812). After the operation, the mice were placed on clean bedding for recovery. Micro-computed tomography (Micro-CT) imaging was performed using a Micro-CT apparatus (Soredex pp1) for in vivo tumor monitoring. The original image stack of representative images is processed with standard ROI tools (On Demand 3D CD Viewer).

#### Syngeneic tumor model

Suspensions of LLC1 lung carcinoma cells were prepared for implantation and washed in PBS. The re-suspended the cells (1 × 10^5^) in 50 µL PBS were mixed with 50 µL Matrigel (Corning, #354230) and subcutaneously injected into both flanks of C57BL/6J mice. Tumor sizes were measured in perpendicular dimensions using calipers. Volumes were estimated using the formula = 0.5 × a × b^2^ (a and b were the long and short diameters of the tumors, respectively). To collect tissues, at the ethical endpoint all tumor-bearing mice were anesthetized with isoflurane inhalation and sacrificed to dissect and photograph tumors.

#### Chronic stress model

Mice were placed in a 50 mL perforated conical tube for 6 h (from 10:00 to 16:00) to restrict free rotation and movement, during which diet and water were prohibited, and sufficient air circulation was provided. Before tumor inoculation, all stressed mice were subjected to a 7-d constraint pretreatment to adapt to these conditions. Then the mice were randomly assigned to a control group or a daily constraint group until the ethical endpoint was reached. After the confinement, release the mouse from the perforated conical tube, clean the conical tube and perform high-pressure steam sterilization. The stress model was performed following the Institutional Animal Care and Use Committee (IACUC) of Dalian Medical University, certificate number is AEE18018.

#### Behavioral paradigms

Behavioral testing was conducted on mice after 3 w of stress treatment, starting from the day after the last stress treatment. Place the mice in the laboratory 60 min before the start of the experiment to adapt to the surrounding environment. The camera recorded the movement of mice and analyzed it using Xeye Aba software (Beijing Macro Ambition S&T Development Co., Ltd.). Each experiment lasts for 5 min. After completion, spray and wipe the testing equipment with 75% ethanol to remove residual odor, urine, and feces from the previous mouse.

#### Open-field test

An open field chamber (50 cm × 50 cm × 35 cm) was divided into a central (25 cm × 25 cm) and external field. Movement of each mouse was monitored for 5 min with a camera and the total distance moved as well as time spent in the center field were recorded.

#### Elevated plus maze test

The maze device was placed 100 cm above the floor and consisted of two open arms (27 cm × 5.5 cm), two closed arms (27 cm × 5.5 cm) and central platform (5.5 cm × 5.5 cm). In each test, the mouse was placed in the central platform. The number of entries in open and closed arms was measured during the subsequent 5 min.

#### Tail suspension test

Mice were suspended by the tail and secured with adhesive tape at 1 cm from the tips of their tails, 30 cm above the floor of the chamber (35 cm × 35 cm × 50 cm). The behavior of experimental animals was measured for 6 min and the total duration of immobility are quantified.

#### Pharmacological studies in mice and lung cancer cells

Mice were injected intraperitoneally (i.p.) with 5 mg/kg OLZ daily (Selleck, S2493) or subcutaneously (s.c.) with 3 mg/kg NE (MCE, HY-13715) daily. Gemcitabine (Selleck, S1714) is intraperitoneally (i.p.) injected into mice once a week at a dose of 50 mg/kg. Sterile 0.1% DMSO in normal saline was used as the vehicle control. In the *Kras*^*LSL−G12D/WT*^ lung cancer model and subcutaneous syngeneic model, treatment was initiated 2 d after tumor-cell inoculation and continued until tissue dissection. Cells were starved for 12 h and then treated with different drugs NE (10 µM; 48 h) (MCE, HY-13715) and H89 (10 µM; 1 h) (MCE, HY-15979) in a culture medium supplemented with 2% FBS.

#### Gene knockdown with shRNA

Knockdown of genes was performed with specific shRNAs delivered using the second-generation packaging system plasmids psPAX2 (Addgene plasmid, #12260) and pMD2.G (Addgene plasmid, #12259). Specific shRNAs as well as psPAX2 and pMD2.G were co-transfected into HEK 293T cells using Lipofectamine 3000 (Invitrogen, L3000015). Supernatants were collected at 48 h and 72 h after transfection. The appropriate cell lines were infected with lentiviruses and selected with puromycin (2 µg/mL, Sigma, P8833). The shRNAs are listed in Table S1.

#### Plasmid construction

The shRNA fragments targeting human and murine CLOCK were inserted into pLKO vector. Plasmids encoding promoter of human CLOCK were generated by PCR amplification and subcloned into pGL3 expression vector. The fidelity of all vectors was confirmed by DNA sequencing. All the primers used for plasmid construction are listed in Table S2.

#### Dual-luciferase reporter assay

HEK 293T cells were plated in 24-well plates and transfected with CLOCK promoter–driven luciferase constructs (pGL3-CLOCK) or control (pGL3-Basic) luciferase constructs under NE treatment. After 24 h, Fluc/Rluc activities were measured using the Dual Luciferase Reporter Assay System (Promega, E1910).

#### Quantitative reverse transcriptase PCR

Total RNA was extracted from tissue samples or cell lines with TRIzol reagent (Invitrogen, #15596018) according to the the manufacturer’s instructions followed by measuring RNA concentration using a Nanodrop Spectrophotometer (Thermo Scientific). The cDNAs were generated using the EasyScript One-Step gDNA Removal cDNA Synthesis SuperMix (Transgen, AE311) and RT-qPCR was performed using the SYBR Green qPCR mix (Vazyme, Q711-02). ACTB was used as the internal control to normalize RNA expression. All primers used in quantitative PCR are given in Table S3.

#### ALDH^+^ cell staining

Performing ALDEFLUOR assay according to the manufacturer’s guidelines (STEMCELL Technologies, #1700). In short, primary syngeneic tumors are digested into a single cell state using collagenase I (Gibco, #17018029) and Dnase I (Merck, #10104159001). 5 × 10^5^ cells were suspended in 1mL ALDEFLUOR assay buffer. Subsequently, 5 µL of activated ALDEFLUOR reagent was added to the sample tube and mixed, and 5 µL of ALDEFLUOR DEAB reagent was added to the negative control tube. After mixing the sample tube, transfer 0.5 mL of the mixture immediately to the negative control tube. Sample and negative control tubes were incubated at 37 °C for 30 min. Collect cells and analyze them using a flow cytometry (Beckman Coulter, CytoFLEX).

#### Sphere formation assay

Prepare single-cell suspension and inoculate it onto a 24 well ultra-low adhesion plate (Corning, #3473), Cultivate in serum-free DMEM/F12 medium (Gibco, C11330500BT) supplemented with 1% methylcellulose (R&D Systems, HSC001), 20 ng/mL epidermal growth factor (Sigma, E9644), 20 mg/mL basic fibroblast growth factor (Peprotech, 100-18B) and 20 µg/mL B27 (Gibco, #17504044). Feed the culture with fresh serum-free DMEM/F12 supplemented with growth factors every other day. Incubate the plate in a 37 ℃ incubator and a humidified incubator containing 5% CO_2_ for about 2 w until spheroids were formed.

For Extreme limiting dilution assay (ELDA), cells enriched from spheroids were seeded into 96-well ultralow attachment plates (Corning, #3474) with sphere medium at density of 64, 32, 16, 8, 4 and 2 cells per well. After 7 d, positive (sphere formation) well numbers in each group were uploaded and calculated in the ELDA website.

#### Enzyme-linked immunosorbent assay (ELISA)

The level of LLC1 syngeneic tumors and serum norepinephrine (Cloud-Clone, CEA907Ge), epinephrine (Cloud-Clone, CEA858Ge), cortisol (Cloud-Clone, CEA462Ge) in mice were determined by ELISA kit in accordance with the manufacturer’s instructions. Add 50 µL each of dilutions of standard, blank and samples into the according wells, respectively. Then, add Detection Reagent A (50 µL) to each well and immediately incubate for 1 h at 37 °C. Next, aspirate the solution and wash with of Wash Solution (350 µL) to each well for 3 times. Add 100 µL of Detection Reagent B working solution to each well, then incubating for 30 min at 37 °C. Next, repeat the wash process for a total of 5 times and add Substrate Solution (90 µL) to each well and incubate for 10 min at 37 °C in dark. After adding Stop Solution (50 µL) to each well, the optical density of each well at 450 nm wavelength was measured immediately. The standard curve was generated with regression analysis, and the concentrations in each sample were calculated according to the standard curve.

#### Cell viability assay

According to the manufacturer’s instructions, cell viabilities were measured using Cell Count Kit-8 (CCK-8, Meilunbio, MA0218). Seeded the needed cells at a density of 2,500 cells per well in a 96 well plate and incubated 12–24 h individually. Then cells were treated with Cisplatin (Selleck, S1166), Carboplatin (Selleck, S1215), Paclitaxel (Selleck, S1150), Pemetrexed (Selleck, S1135), Gemcitabine (Selleck, S1714) for 48 h. Subsequently, add CCK-8 solution (10 µL) to each well and incubate for 2 h. Measure absorbance at 450 nm using a spectrophotometer.

#### Colony formation

Inoculate human lung cancer cells NCI-H1299 and mouse lung cancer cells LLC1 at a cell density of 2,000 cells per dish into a 60 mm plastic culture dish and allow them to grow for 10–14 d until clones are visible. After washing the cells with PBS, they were fixed with 4% paraformaldehyde and stained with 0.4% crystal violet (Sangon biotech, A100528). Three stained colony images were counted using Image J.

#### Western blotting

Use an appropriate amount of protein lysate to lyse cells (50 mM Tris, pH 7.5, 120 mM NaCl, 1% Triton X-100, 0.5% sodium deoxycholate, 0.1% SDS, 5 mM EDTA reagent with a protease inhibitor cocktail (MCE, HY-K0010) or phosphatase inhibitor cocktail (Bimake, B15001) on ice for 30 min. Lysates were centrifuged at 12,000 × g for 15 min at 4 °C to obtain protein samples. Proteins were separated by 10% SDS polyacrylamide gel electrophoresis (SDS-PAGE) and then transferred to NC membranes (Merck Millipore, HATF00010) by wet blotting. Nonspecific binding sites on the membranes were blocked by incubation with 5% non-fat milk (Sangon biotech, A600669) or 5% BSA (Sigma-Aldrich, V900933) on a shaker for 1 h. Membranes were then incubated with primary antibodies for 12 h and subsequently incubated with HRP-conjugated secondary antibodies (Thermo Fisher Scientific) for 1 h. Finally, protein bands were visualized using ultra-sensitive ECL detection (Thermo Fisher Scientific, #34580) and quantified using a ChemiDoc MP imaging system (Bio-Rad). Antibodies used for western blotting were: NANOG (Gene Tex, GTX100863), Sox2 (Santa Cruz Biotechnology, sc-365,823), CD166 (Proteintech, 21972-1-AP), OCT4 (Gene Tex, GTX101497), CLOCK (Abcam, ab3517), ADRB2 (Proteintech, 13096-1-AP), CREB (Proteintech, 12208-1-AP), p-CREB (Proteintech, 28792-1-AP), PKA (Proteintech, 55382-1-AP) and β-Actin (Proteintech, 66009-1-lg). Full length western blots were listed in Supplementary Materials.

#### Immunofluorescence

Fix cells with 4% paraformaldehyde, then permeate the membrane with 0.4% TritonX-100 in PBS, and seal with a blocking solution (1% BSA; Sigma, V900933) at room temperature for 2 h. Primary antibodies against c-Fos (Abcam, ab208942) and TH (Proteintech, 25859-1-AP) were incubated overnight at 4 °C followed by incubation with secondary antibodies for 1 h at room temperature. The samples ware re-stained with 4 ‘, 6-diamino-2-phenylindole dihydrochloride (DAPI) (Sigma Aldrich, D9542) and fixed on a glass slide with anti-quenching medium. Perform image acquisition and analysis in a confocal microscope (Leica, TCS SP5II).

#### Immunohistochemical staining

Immunohistochemical staining was performed using standard methods, in which tissue samples were embedded in paraffin and then dewaxed with citric acid (pH 6.0) and rehydrated for 2 min for antigen recovery. Block endogenous catalase in H_2_O with 3% hydrogen peroxide for 20 min, and block tissue samples with 10% goat serum for 30 min. The antibodies CD166 (Proteintech, 21972-1-AP), Ki67(Abcam, ab15580), CLOCK (Abcam, ab3517) were incubated overnight at 4 °C and then incubated with HRP peroxidase-bound secondary antibody at 37 °C for 1 h. Stain the tissue samples using DAB (ZSGB-BIO, ZLI-9018) and wash with water to terminate the reaction. Identify the cell nucleus using hematoxylin (Coolaber’s, SL7050) staining for 3–5 min, then rinse with water. Finally, dehydrate the tissue and seal it with neutral resin. The second antibody bound to HRP peroxidase comes from mouse primary tissue staining and IHC assay kit (ZSBIO, pv-9002). Select six random regions to quantify the positive levels of each tumor tissue slice, and use Image J to quantify the values of each image. For patient’s sample, the overall positivity scores for the target protein were calculated as H-scores = 1 × (percentage of weak intensity area) + 2 × (percentage of moderate intensity area) + 3 × (percentage of strong intensity area) across the entire stained sample.

#### Hematoxylin eosin staining

Hematoxylin eosin (H&E) staining is used to determine the proliferation of mouse tumor tissue cells. Place tumor tissue paraffin sections in xylene I for 20 min, anhydrous ethanol I for 20 min, anhydrous ethanol II for 5 min, and 75% ethanol for 5 min, and then rinse with tap water. The nucleus and cytoplasm are stained sequentially. Finally, the slices were dehydrated and fixed with neutral gum, followed by microscopic examination, image acquisition, and analysis. Select six random regions to quantify the positive levels of each tumor tissue slice, and use Image J to quantify the values of each image.

#### ChIP assay

According to the manufacturer’s protocol, use the ChIP-IT Express chromatin immunoprecipitation assay kit (Active Motif, #53008) for chromatin immunoprecipitation determination. In short, incubate the cells with a fixed solution at room temperature on a shaker for 10 min, crosslink histones with DNA, and then wash in cold 1×PBS. Add glycine termination fixation solution to the plate for 10 s to stop the fixation reaction. Cells were then scraped off by scraping with the cell scraping solution including PMSF (final concentration is at 0.6 mM) and centrifuged at 2,500 × g for 10 min at 4 °C. Incubate the resuspended cells with 1mL of pre-cooled lysis buffer and incubate on ice for 30 min. Transfer the cells to a pre-chilled dounce homogenizer and agitated 10–20 times on ice to facilitate nuclear release. DNA was sheared with a sonicator followed by centrifugation at 15,000 × g for 10 min at 4 °C and 10 µL was carefully transferred as “input DNA” at -20 °C. The remaining chromatin was immunoprecipitated with 5 µg CREB antibody (Proteintech, 12208-1-AP) or a corresponding control IgG antibody (Proteintech, 10284-1-AP) and protein A/G magnetic beads (Invitrogen, 10001D) at 4 °C overnight. Beads were washed with ChIP buffer I and ChIP buffer II and resuspended in 50 µL elution buffer AM2. Chromatin was eluted with 50 µL of reverse crosslinking buffer and incubated with 2 µL proteinase K (Solarbio, P9460) at 37 °C for 1 h. Finally, DNA was collected with 2 µL Proteinase K stop solution by centrifugation and used for subsequent PCR analysis. All primers used in quantitative PCR are given in Table S3.

#### Clinical specimens

Blood specimens, Hospital anxiety and depression scale (HADS) questionnaires regarding anxiety and depression were collected from 59 lung cancer patients who were examined and treated in the First Affiliated Hospital of Dalian Medical University. Fresh specimens were stored in a -80 °C freezer until use. All specimens were collected in compliance with the informed consent policy.

#### RNA-seq

Total RNA from NCI-H1299 cells treated with NE for 48 h was extracted with TRIzol reagent (Invitrogen, #15596018). Libraries were prepared by Novogene (Beijing, China) using the NEBNext UltraTM RNA Library Prep Kit for Illumina (NEB), as the manufacturer’s instructions. All samples were sequenced using the Illumina Hiseq platform with a reading length of 125 bp at both the 5’ and 3’ ends. Based on standard default parameters, map RNA-seq data was used to reference the genome at human hg38 using Hisat2 v2.0.5 software. The expected number of each thousand base segments of transcriptional sequence per million base pair sequencing was based on the length of the gene and the read count mapped to the gene through the feature count to estimate expression level of each gene (v1.5.0-p3). Differentially expressed genes (DEG) were analyzed using the DEseq2R package. The adjusted *P* value of < 0.05 and | log2 (foldchange) | ≥ 1 was designated as differentially expressed. Gene ontology (GO) enrichment analysis of DEGs was performed using the cluster archiver R package software (3.8.1). These sequence data have been submitted to the GenBank databases under accession number GSE268860.

### Statistical analyses

Use SPSS software (version 16.0) or GraphPad Prism 6.0 for statistics. As shown in the legend, use Student’s t-test (two tailed unpaired), one-way ANOVA test statistical comparison or likelihood ratio test. The tumor growth curve data was analyzed using one-way ANOVA test statistical comparison at the ethical endpoint. Use Kaplan-Meier plots to estimate the overall survival (OS), post progression survival (PPS), and first progression (FP) of cancer patients. Use logarithmic rank test to test the significance of differences in survival rates. The risk ratio and 95% confidence interval for each variable were estimated. Correlation analysis was evaluated using Pearson correlation test.

## Results

### OLZ suppresses chronic stress-induced lung tumorigenesis

**Fig. 1:**
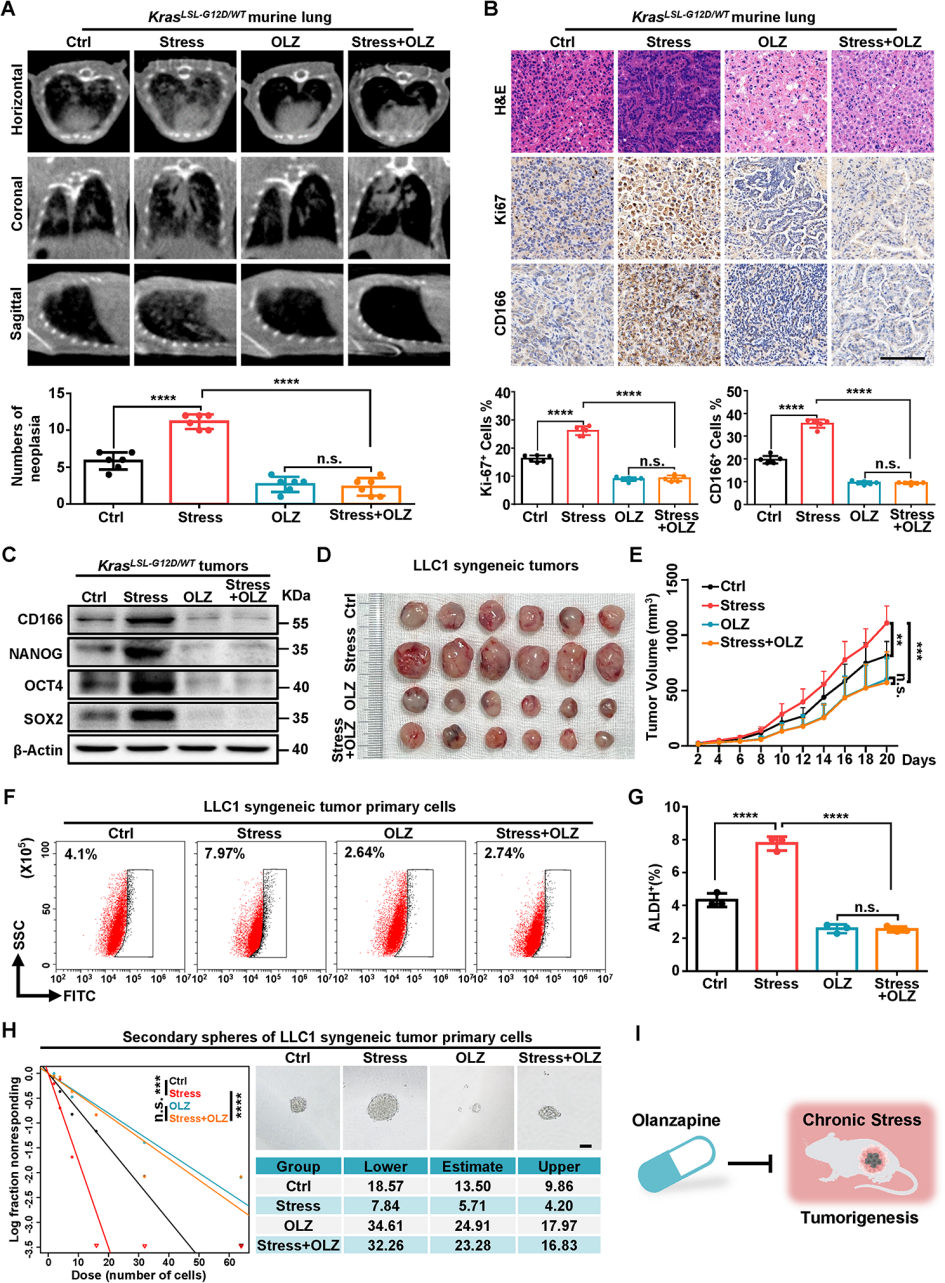
OLZ suppresses chronic stress-enhanced lung tumorigenesis. (**A**) Representative horizontal, coronal and sagittal micro-CT scans (up) and quantification (bottom) of neoplasia in vehicle control (i.p., 0.1% DMSO in normal saline) or olanzapine (OLZ, i.p., 5 mg/kg/d) treated ctrl and stress *Kras*^*LSL−G12D/WT*^ mice at 7 w after AAV-GFP-Cre injection. (**B**) Representative typical H&E and IHC staining and quantification of the indicated markers of the *Kras*^*LSL−G12D/WT*^ murine lung in (**A**). Scale bars, 100 μm. (**C**) Relative protein levels of cancer stemness factors in *Kras*^*LSL−G12D/WT*^ tumors with or without OLZ in Ctrl or Stress treated. β-Actin was used as a loading control. (**D, E**) Representative tumor image (**D**) and growth curve (**E**) of Ctrl, Stress, OLZ and Stress with OLZ treated LLC1 syngeneic tumor in mice. (**F, G**) Flow cytometry analysis (**F**) and populations (**G**) for ALDH-positive cells in LLC1 syngeneic tumor primary cells from tumors in (**D**). (**H**) Left: second generation Extreme Limiting Dilution Analysis (ELDA) was performed by plating spheroids of LLC1 syngeneic tumor primary cells from tumors in (**D**). Top right: The representative sphere images are shown. Bottom right: Stemness frequency of primary cells. Spheres were counted from 16 replicate wells. Scale bars, 100 μm. (**I**) Model of OLZ suppresses chronic stress-enhanced lung tumorigenesis. Data in A, B and E represent the mean ± SEM (*n* = 6 mice each group). Data in G represent the mean ± SEM (*n* = 3 independent experiments). Statistical significances were determined using one-way ANOVA followed by Sidak’s multiple comparison test (**A, B, E, G**) or likelihood ratio test (**H**). (** *P* < 0.01, *** *P* < 0.001, **** *P* < 0.0001, n.s. *P* > 0.05)

To investigate whether olanzapine (OLZ) could reverse the promotion of tumorigenesis under chronic stress, *Kras*^*LSL−G12D/WT*^ mice were intratracheal injected AAV-GFP-Cre and randomly divided into Ctrl, Stress, OLZ, and Stress with OLZ group. Seven weeks after virus injection, the mice were scanned with Mirco-CT and horizontal, coronal and sagittal images were performed. The numbers of neoplasia showed that significant glassy nodules occurred in the lungs of mice in the Stress group while slight glassy nodules in Stress with OLZ group (Fig. 1A). The positive rates of Ki67 and CD166 in IHC staining were scored and showed that Ki67 and CD166 in the Stress group were significantly increased compared with Ctrl groups, while were significantly reduced in Stress with OLZ group (Fig. 1B). The protein expression of CD166, NANOG, OCT4 and SOX2 stemness-associated factors were higher in stress group while significantly lower in Stress with OLZ groups in the *Kras*^*LSL−G12D/WT*^ tumors (Fig. 1C).

To further investigate the OLZ effect in reversing chronic-enhanced tumor progression, we set out mouse subcutaneous tumor model with Lewis lung carcinoma (LLC1) cells in C57BL/6J mice under Ctrl, Stress, OLZ, and Stress with OLZ treatment. Stress treatment significantly increased tumor volume compared with the Ctrl group while the OLZ treatment reversed the tumor volume in Stress with OLZ group (Fig. 1D, E). Similarly, the protein and mRNA level of CD166, NANOG, OCT4 and SOX2 were increased in Stress group and were reduced under OLZ treatment (Fig. S1A-C). To further verify the effect of OLZ in reversing the stemness of tumor cells enhanced by chronic stress, primary cells were extracted from LLC1 tumor tissues in the four groups and stained with ALDH. The proportion of ALDH^+^ primary LLC1 cells in the Stress group was significantly higher while OLZ treatment decreased the enhancing effect of chronic stress on ALDH^+^ cells in primary LLC1 cells (Fig. 1F, G). The sphere formation capacity of primary cells was increased in Stress group and OLZ treatment reduced the sphere formation ability under stress both in the primary and secondary spheres (Fig. 1H and S1D). These results indicate that OLZ suppresses chronic stress-enhanced lung tumorigenesis (Fig. 1I).

### OLZ relieves chronic stress-induced both mPFC activity-NE releasing and depression-like behavior in mice with lung cancer

**Fig. 2:**
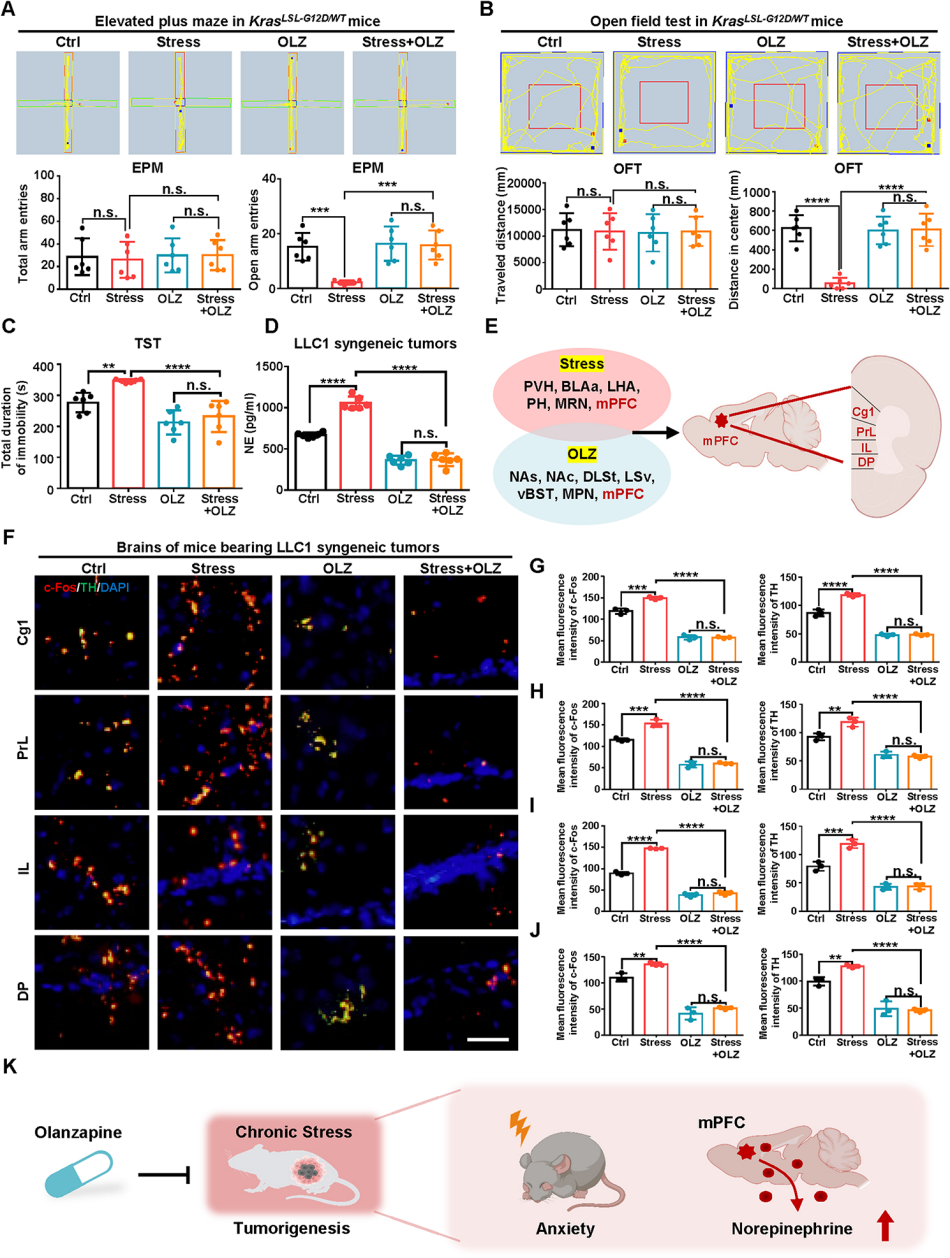
OLZ relieves chronic stress-induced both mPFC activity-NE releasing and depression-like behavior in mice with lung cancer. (**A**) Representative typical image of elevated plus maze (EPM) (top) and quantification of total arm entries (bottom left) and open arm entries (bottom right) in *Kras*^*LSL−G12D/WT*^ mice treated with vehicle control (i.p., 0.1% DMSO in normal saline) or olanzapine (OLZ, i.p., 5 mg/kg/d) under Ctrl and stress at 7 w after AAV-GFP-Cre injection. (**B**) Representative typical image of open field test (OFT) (top) and quantification of traveled distance (bottom left) and distance in center (bottom right) of *Kras*^*LSL−G12D/WT*^ mice in (**A**). (**C**) Total duration of immobility in tail suspension test of *Kras*^*LSL−G12D/WT*^ mice in (**A**). (**D**) NE concentration of LLC1 tumors subcutaneously inoculated in C57BL/6J mice treated with vehicle control (i.p., 0.1% DMSO in normal saline) or OLZ (i.p., 5 mg/kg/d) under Ctrl and stress. (**E**) Overlapping the brain regions related in stress and OLZ and showed the common mPFC region. (**F**) Representative typical immunofluorescence image of c-Fos and tyrosine hydroxylas (TH) in cingulate cortex (Cg1), prelimbic cortex (PrL), infralimbic corex (IL) and dorsopeduncular cortex (DP) regions of mice bearing LLC1 syngeneic tumors in (**D**). Scale bars, 20 μm. (**G-J**) Quantification of cFos and TH stain in Cg1, PrL, IL and DP regions in (**E**). (**K**) Model of OLZ relieved chronic stress-induced depression behavior and mPFC activity-NE releasing in lung cancer. Data in A, B, C, D represent the mean ± SEM (*n* = 6 mice each group). Data in G-J represent the mean ± SEM (*n* = 3 independent experiments). Statistical significances were determined using one-way ANOVA followed by Sidak’s multiple comparison test (** *P* < 0.01, *** *P* < 0.001, **** *P* < 0.0001, n.s. *P* > 0.05)

To investigate the behavioral effect of OLZ in rescuing stress-enhanced tumorigenesis, we set out elevated plus maze and open field test to test the anxiety-like behavior of the *Kras*^*LSL−G12D/WT*^ mice in the Ctrl, Stress, OLZ and Stress with OLZ group. The mice in the four groups showed no significant differences in total arm entries indicating no differ in spontaneous mobility. However, the mice in Stress group showed decreased open arm entries, indicating anxiety-like behavior while the OLZ treatment rescued the behavior in elevated plus maze (Fig. 2A). Meanwhile, the mice in Stress group showed decreased distance in center while the OLZ treatment rescued the anxiety-like behavior with equal traveled distance (Fig. 2B). Besides, the mice in Stress group showed increased total duration of immobility while the OLZ treatment rescued the depression-like behavior with decreased immobility duration (Fig. 2C). Similar results were also found in the LLC1 subcutaneous tumor model (Fig. S2A-C). Furthermore, the level of NE, epinephrine and cortisol were higher under stress while OLZ treatment reduced the stress-induced increase of NE but had no effect on epinephrine or cortisol in the LLC1 syngeneic tumor (Fig. 2D, S2D and S2E). Similar results were also found in the serum level of mice with LLC1 subcutaneous tumor (Fig. S2F-H). These results indicated that OLZ rescued chronic stress-induced anxiety-like behavior in *Kras*^*LSL−G12D/WT*^ lung cancer model and LLC1 syngeneic tumor model.

To further investigate the brain region-specific effects of OLZ under chronic stress, we selected the brain regions activated under chronic stress and OLZ treatment and analysed the sympathetic neuronal activation in the subregion of mPFC (Fig. 2E) [22–25]. Stress increased the expression of both the c-Fos expression and tyrosine hydroxylase (TH) in cingulate cortex (Cg1), prelimbic cortex (PrL), infralimbic corex (IL) and dorsopeduncular cortex (DP) subregion of mPFC while OLZ treatment suppressed the stress-enhanced the expression of c-Fos and TH (Fig. 2F-J) [5]. These results demonstrated that OLZ relieved chronic stress-induced anxiety-like behavior and mPFC activity -NE releasing in mice with lung cancer (Fig. 2K).

### Circadian gene CLOCK mediates NE-induced tumor growth and cancer stemness

**Fig. 3:**
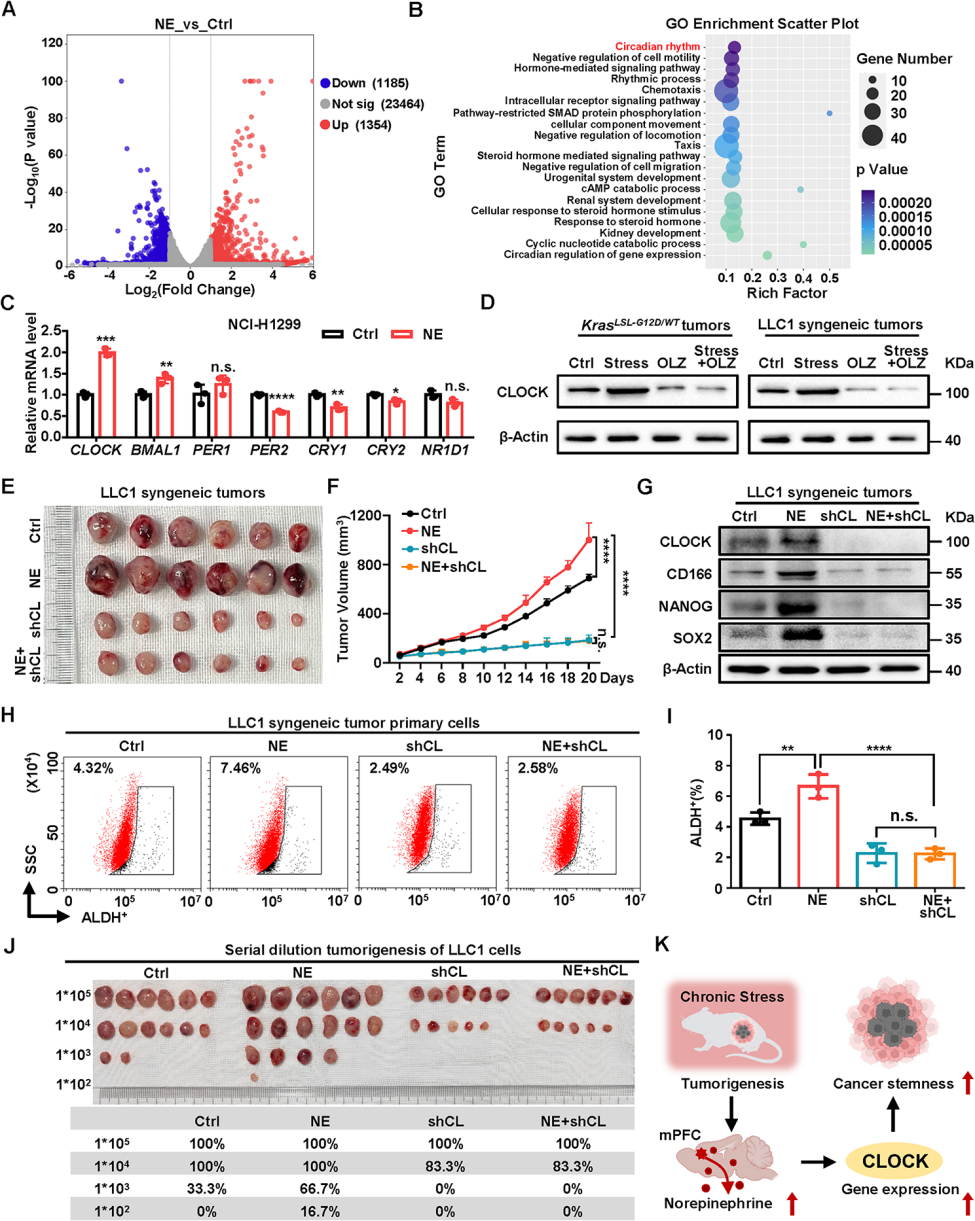
Circadian gene CLOCK mediates NE-induced tumor growth and cancer stemness. (**A**) Volcano plots represent differentially expressed genes (DEGs) of NCI-H1299 cells treated with NE (10 µM, 48 h). The number of significantly variant genes (fold change > 2, *P* value < 0.05) was shown. Vertical dashed lines indicate cut-off of fold change (2), whereas the horizontal dashed lines indicate cut-off of *P* value (0.05). (**B**) Scatter plot of Gene ontology (GO) enrichment from upregulated genes of NE treated NCI-H1299 cells. (**C**) Relative mRNA levels of core circadian genes in NCI-H1299 cells treated with NE (10 µM, 48 h). (**D**) Relative protein levels of CLOCK in *Kras*^*LSL−G12D/WT*^ tumors and LLC1 syngeneic tumors. β-Actin was used as a loading control. (**E, F**) C57BL/6J mice were subcutaneously inoculated with LLC1 cells with CLOCK silencing (shCL) and supplement of NE (s.c., 3 mg/kg/d) (**E**) and tumor volumes were monitored (**F**). (**G**) Relative protein levels of CLOCK and stemness factors of the tumors in (**E**). β-Actin was used as a loading control. (**H, I**) Flow cytometry analysis (**H**) and populations (**I**) for ALDH-positive cells in LLC1 syngeneic tumor primary cells from tumors in (**E**). (**J**) Serially diluted tumor cells from syngeneic tumors (**E**) were subcutaneously inoculated at 4 different sites into each group of mice. Statistical analysis of tumorigenesis with indicated cell numbers and different treatments is shown. (**K**) Model of NE enhanced CLOCK to promote cancer stemness in lung cancer. Data in C, I represent the mean ± SEM (*n* = 3 independent experiments). Data in F represent the mean ± SEM (*n* = 6 mice each group). Statistical significances were determined using two tailed unpaired Student’s t test (**C**) or one-way ANOVA followed by Sidak’s multiple comparison test (**F, I**) (* *P* < 0.05, ** *P* < 0.01, *** *P* < 0.001, **** *P* < 0.0001, n.s. *P* > 0.05)

To determine the mechanisms by which chronic stress promoted tumorigenesis, we performed RNA-Seq with NCI-H1299 treated with NE. Comparison between Ctrl and NE-treated cells identified 1354 genes significantly upregulated by NE (Fold change > 2, *p* < 0.05) (Fig. 3A). GO analysis on the NE upregulated genes revealed that the circadian rhythm process ranked the top enriched pathways (Fig. 3B). We determined the key core circadian genes responsible for NE-induced tumorigenesis and found that *CLOCK* mRNA level was significantly increased in NE treated NCI-H1299 and LLC1 cells (Fig. 3C and S3A). CLOCK protein expression was also elevated in a time-dependent manner of NE in NCI-H1299 and LLC1 cells (Fig. S3B). We verified that OLZ rescued the stress-induced CLOCK protein expression in both *Kras*^*LSL−G12D/WT*^ tumors and LLC1 syngeneic tumors (Fig. 3D).

To further verify the role of CLOCK in NE-induced tumorigenesis, we determined the effects of CLOCK deficiency on tumor growth in LLC1 syngeneic mouse model undergoing NE treatment. The results showed that the CLOCK deficiency reversed the NE treatment increased tumor volume (Fig. 3E, F). Importantly, ablation of CLOCK significantly restored NE-elevated stemness related factors (Fig. 3G and S3C). We harvested primary tumor cells and found that CLOCK deficiency substantially reversed the NE-elevated ALDH^+^ subpopulations (Fig. 3H, I) and inhibited the diameter, number, and capacity of sphere formation in LLC1 syngeneic tumor primary cells (Fig. S3D-F). We also performed reimplantation essay with the serial dilutions of LLC1 cells in C57BL/6J mice. The tumor formation rate of LLC1 cells (1 × 10^3^) in mice treated with NE was significantly increased to 66.7% while the rate of LLC1 cells with CLOCK deficiency decreased to 0%. The result showed that CLOCK knock down inhibited the NE-enhanced tumor formation rates (Fig. 3J). Taken together, our finding indicates that circadian gene CLOCK mediated NE-induced tumor growth and cancer stemness (Fig. 3K).

### NE promotes CLOCK transcription by activating ADRB2-PKA-CREB pathway

**Fig. 4:**
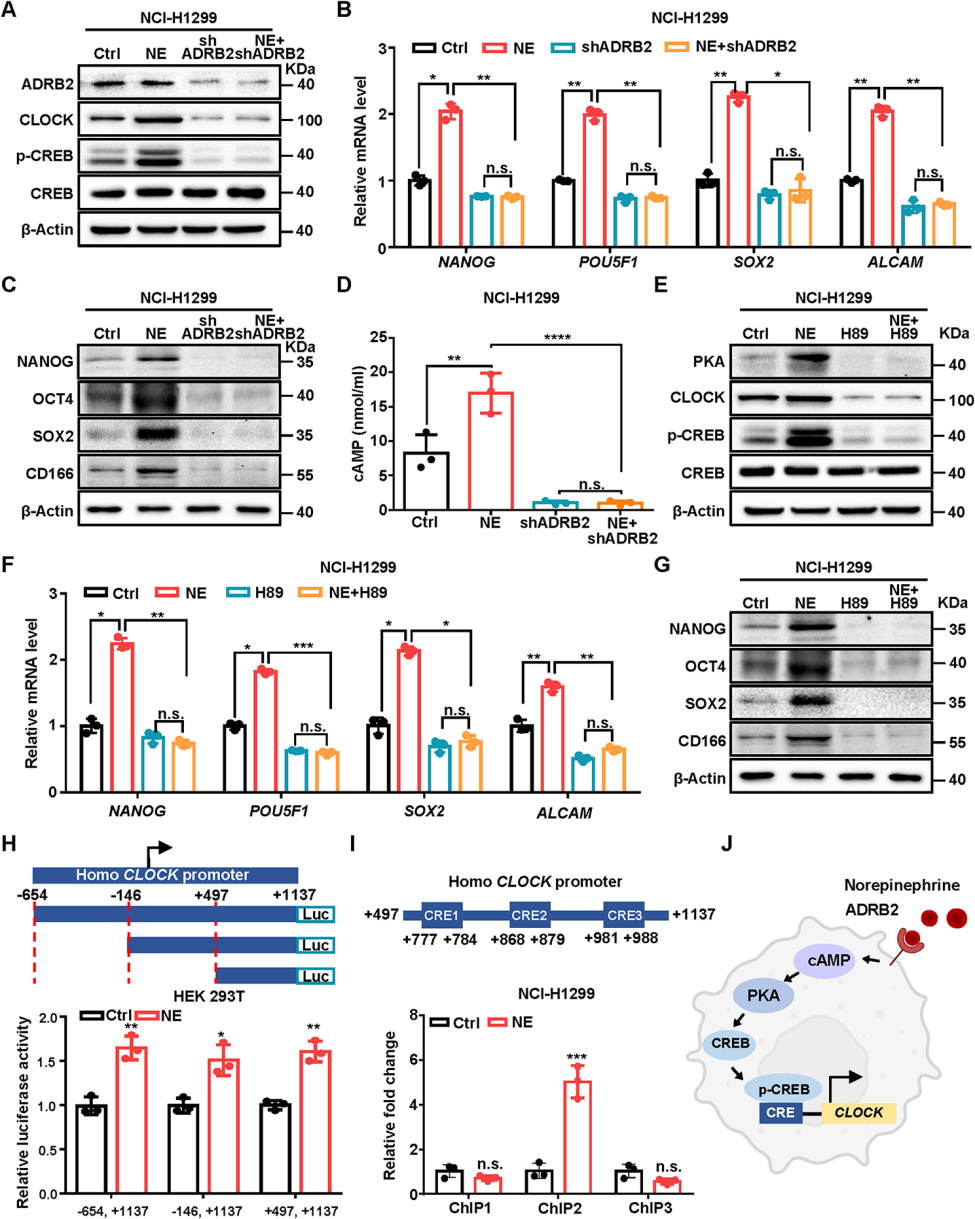
NE promotes CLOCK transcription by activating ADRB2-PKA-CREB pathway. (**A-D**) Relative indicated protein levels (**A**), mRNA levels (**B**) and protein levels (**C**) of stemness factor and cAMP concentration (**D**) in the NCI-H1299 cells with ADRB2 deficiency (shADRB2) and NE supplement (10 µM, 48 h). β-Actin was used as a loading control. (**E-G**) Relative indicated protein levels (**E**), mRNA levels (**F**) and protein levels (**G**) of stemness factor in the NCI-H1299 cells with NE (10 µM, 48 h) and H89 (10 µM, 1 h). β-Actin was used as a loading control. (**H**) Relative luciferase activity of truncated CLOCK promoters in HEK 293T cells under NE treatment (10 µM, 48 h). (**I**) Relative fold change of ChIP-qPCR primer targeting putative CREB binding motifs on CLOCK promoter in NCI-H1299 cells treated with NE (10 µM, 48 h). (**J**) Model of NE stimulates ADRB2-PKA-CREB pathway to transactivate CLOCK. All data represent the mean ± SEM (*n* = 3 independent experiments). Statistical significances were determined using one-way ANOVA followed by Sidak’s multiple comparison test (**B, D, F**) and two tailed unpaired Student’s t test (**H, I**) (* *P* < 0.05, ** *P* < 0.01, *** *P* < 0.001, n.s. *P* > 0.05)

NE is one of the critical stress-related neurotransmitters that activates ADRB-PKA-CREB for transactivate its target genes [10]. To further identify the mechanism of chronic stress-induced NE modulates circadian clock to promote cancer stemness, we established ADRB2 stable knockdown cells by shRNAs [26] in NCI-H1299 cells and found that ADRB2 deficiency reversed NE-induced CLOCK and stemness-related factors NANOG, OCT4, SOX2 and CD166 level (Fig. 4A-C). We then measured cAMP levels and found that depletion of ADRB2 significantly reversed the NE-elevated cAMP level (Fig. 4D). Next, we measured the role of enzymatic activation of cAMP dependent kinase PKA in NE-induced stem-like properties. We observed that CLOCK and stemness-related factors were highly activated in NE-treated cells could be abolished by the PKA inhibitor, H89 (Fig. 4E-G). To investigate whether CREB transcriptionally regulate CLOCK expression, we analyzed data from the Cistrome Data Browse [27, 28] to confirm CREB’s occupancy at the promoter region of CLOCK in A549 cells [29]. Further, luciferase reporter assay revealed that the + 496 to + 1137 region on *CLOCK* promoter was required for NE-induced *CLOCK* transactivation (Fig. 4H). As putative motifs were located in CREB responsive region on *CLOCK* promoter, we conducted chromatin immunoprecipitation (ChIP)-qPCR and identified CREB-2 (+ 868 to + 879) as the binding site of CREB (Fig. 4I). These results indicated that NE stimulates ADRB2-PKA-CREB pathway to transactivate CLOCK (Fig. 4J).

### Ablation of CLOCK or OLZ restrains chronic stress-/NE-induced gemcitabine resistance in lung cancer

**Fig. 5:**
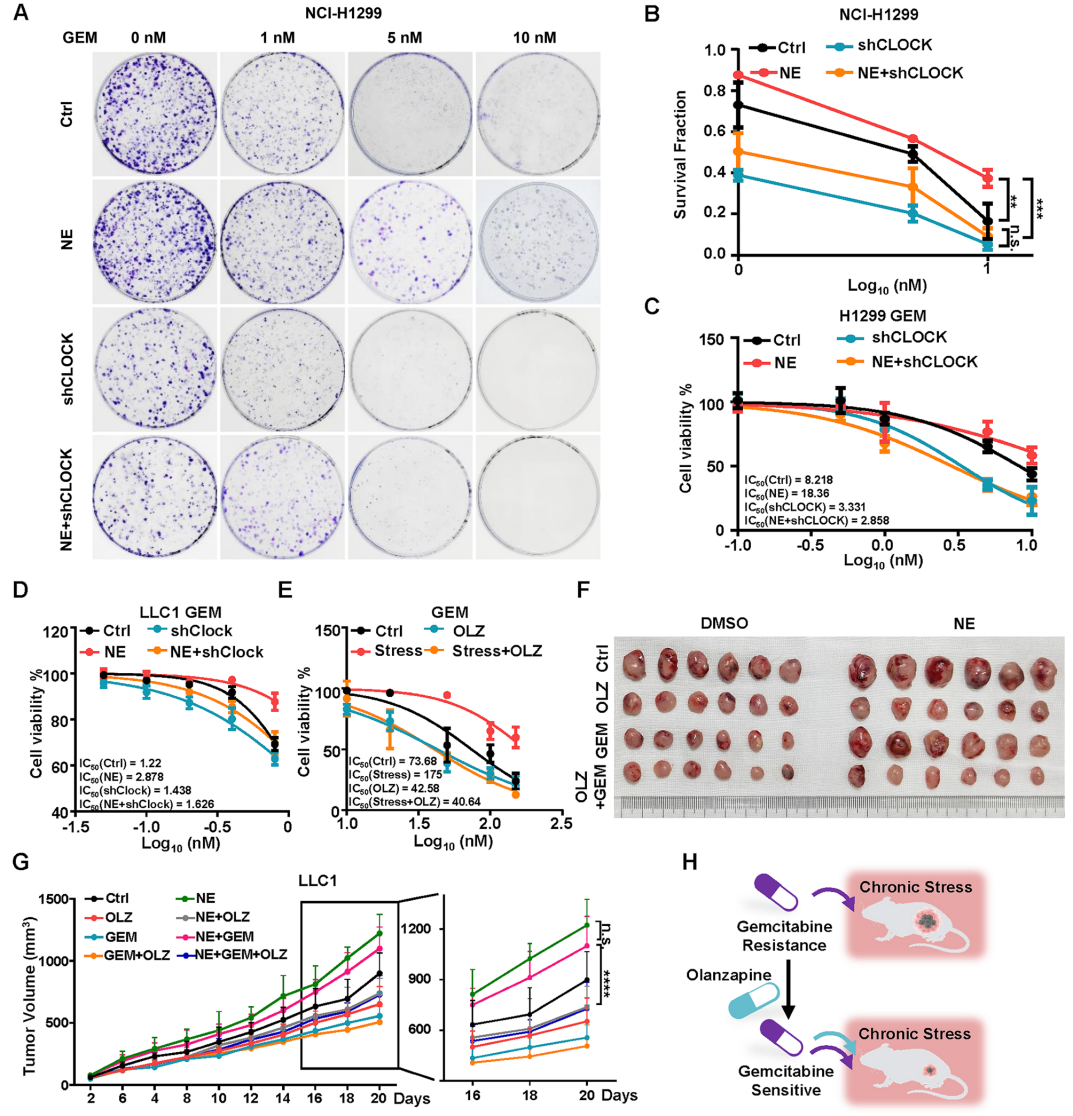
Ablation of CLOCK or OLZ restrains chronic stress-/NE-induced gemcitabine resistance in lung cancer. (**A, B**) NCI-H1299 cells followed depletion of CLOCK (shCLOCK) and supplement of NE then treated with 0, 1, 5 and 10 nM gemcitabine (GEM). The proliferation of NCI-H1299 cells were determined by assessing the area of colonies stained with crystal violet. (**C**) NCI-H1299 cells followed depletion of CLOCK (shCLOCK) and supplement of NE then treated with different concentrations of GEM for 48 h. Cell viability was determined by CCK-8 assay. The numbers in the figure keys represent the concentrations (nM) of GEM. Cells treated with vehicle serve as a blank control. (**D**) Cell viability of LLC1 cells followed depletion of CLOCK (shClock) and supplement of NE then treated with different concentrations of GEM. (**E**) Cell viability of primary LLC1 cells in the determined groups treated with different concentrations of GEM. (**F, G**) C57BL/6J mice were subcutaneously inoculated with LLC1 cells and supplement of NE (s.c., 3 mg/kg/d), OLZ (i.p., 5 mg/kg/d) or GEM (i.p., 50 mg/kg/w) (**F**), and tumor volumes were monitored (**G**). (**H**) Model of OLZ enhanced the sensitivity of lung cancer cells to chemotherapy under chronic stress. Data in B represent the mean ± SEM (*n* = 3 independent experiments). Data in C, D, E represent the mean ± SEM (*n* = 5 independent experiments). Data in G represent the mean ± SEM (*n* = 6 mice each group). Statistical significance was determined using one-way ANOVA followed by Sidak’s multiple comparison test (** *P* < 0.01, *** *P* < 0.001, **** *P* < 0.0001, n.s. *P* > 0.05)

Chronic stress-induced NE caused resistance of oral or non-small cell lung cancer cells to cisplatin [30, 31], we investigated the effect of CLOCK deficiency on the sensitivity of NCI-H1299 to chemotherapy by measuring the viability and proliferation of the cells subjected to NE treatment. Ablation of CLOCK restored the sensitivity of the NE-treated cells to gemcitabine (GEM), whereas NE-induced carboplatin (CBP), cisplatin (CDDP), pemetrexed (PEM) or paclitaxel (PTX) chemoresistance cannot be reversed by CLOCK deficiency (Fig. 5A-C and S4A). Similarly, CLOCK knock down re-sensitize the LLC1 cells to GEM under NE treatment (Fig. 5D and S4B-D). Since olanzapine is currently administered to cancer patients for the purpose of preventing nausea and vomiting induced by chemotherapy [19], we wonder if olanzapine modulates the sensitivity of cancer cells to chemotherapeutic agents. OLZ treatment reinstated the sensitivity of LLC1 syngeneic tumor primary cells to GEM under chronic stress treatment but had little effect on CBP, CDDP, PEM or PTX (Fig. 5E and S4E). Furthermore, combination OLZ and GEM markedly inhibited the tumor growth and restored the sensitivity of GEM under NE treatment (Fig. 5F, G). These results confirmed that ablation of CLOCK or OLZ restrained chronic stress-/NE-induced gemcitabine resistance in lung cancer (Fig. 5H).

### CLOCK expression is positively associated with depression status, serum NE level and poor prognosis in lung cancer patients

**Fig. 6:**
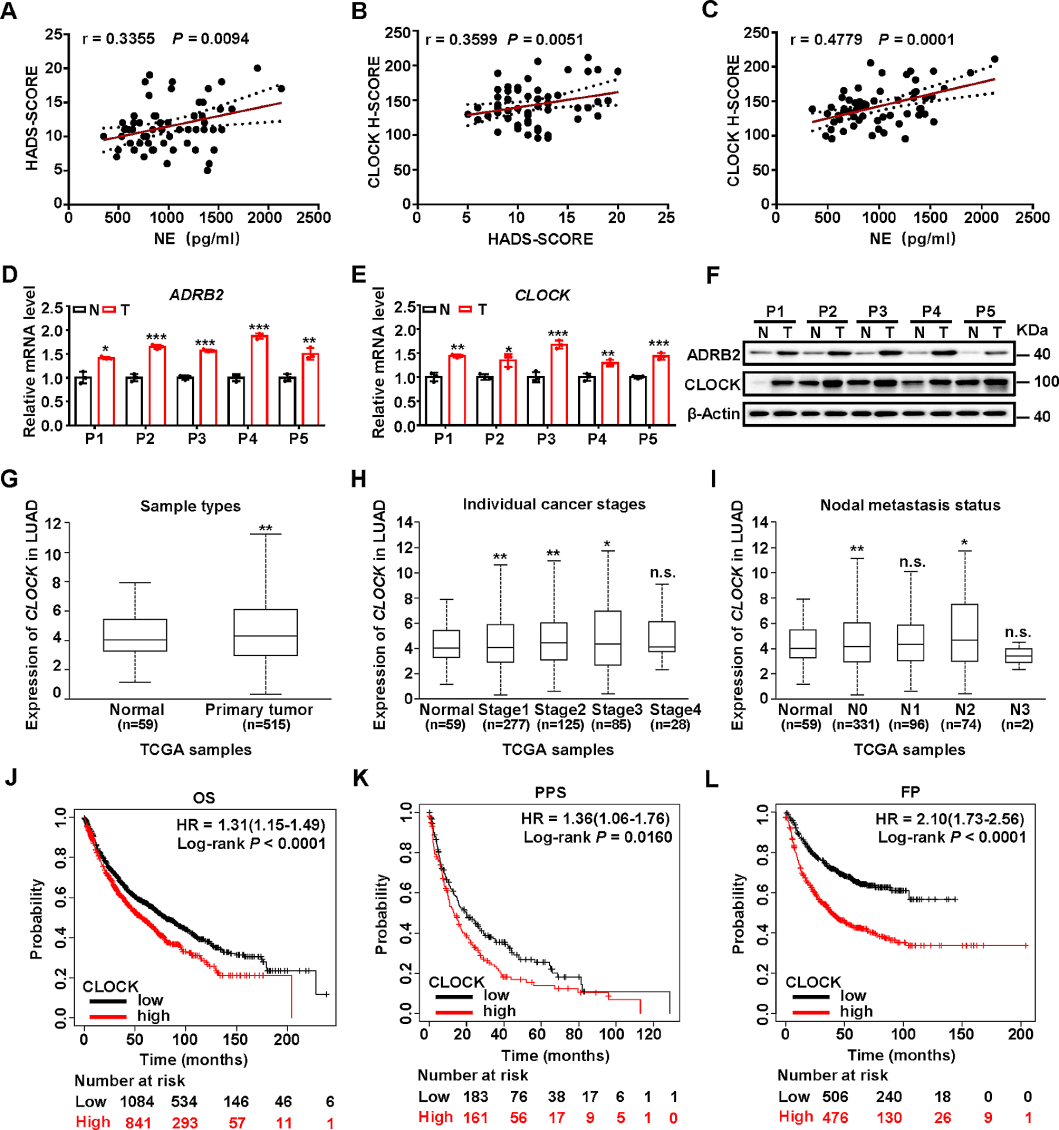
Clinical relevance of NE and CLOCK in patients with lung cancer CLOCK expression is positively associated with depression status, serum NE level and poor prognosis in lung cancer patients. (**A**) Linear regression analysis of the correlations between serum NE concertation and Hospital Anxiety and Depression Scale (HADS) score assessed by HADS questionnaires (*n* = 59 biological replicates). (**B, C**) Linear regression analysis of the correlations between CLOCK score of lung tissues in patients with lung cancer and HADS score (**B**) and serum NE concertation (**C**) (*n* = 59 biological replicates). (**D, E, F**) Relative mRNA levels of *ADRB2* (**D**), *CLOCK* (**E**) and protein levels (**F**) in adjacent normal tissues (N) and tumor tissues (T) in patients with lung cancer. β-Actin was used as a loading control. (**G, H, I**) Box plot comparison of *CLOCK* expression in lung adenocarcinoma (LUAD) according to sample types (**G**), individual cancer stages (**H**) and nodal metastasis status (**I**). These results are based upon data generated by the UALCAN Network. (**J, K L**) Kaplan–Meier overall survival (OS) (**J**), post progression survival (PPS) (**K**) and first progression (FP) plots (**L**) of lung cancer patients created using Kaplan–Meier Plotter network. Patients were classified into CLOCK high and CLOCK low subgroups and analyzed as indicated. Data in D, E represent the mean ± SEM (*n* = 3 independent experiments). Statistical significances were determined using Pearson (r) correlation (**A-C**), two tailed unpaired Student’s t test (**A-E, G-I**) or Log-rank tests (**J-L**) (* *P* < 0.05, ** *P* < 0.01, *** *P* < 0.001, n.s. *P* > 0.05)

To evaluate the correlations between stress level, serum NE and CLOCK in patients with lung cancer, we performed prospective analysis of 59 lung cancer cases. We first evaluated subjective stress level scored by Hospital Anxiety and Depression Scale (HADS) and showed that serum NE levels were positive associated with HADS-score (Fig. 6A). Further, we demonstrated that CLOCK H-score assessed by IHC analysis was positive associated with HADS score and NE serum level (Fig. 6B, C). Further, surgically collected 5 pairs of lung cancer tissues displayed higher mRNA and protein levels of ADRB2 and CLOCK and mRNA level of stemness-related factors than their adjacent normal tissues (Fig. 6D-F and S5A-C).

To evaluate the clinical relevance of CLOCK in lung cancer patients, we validated the expression of CLOCK and predicted prognosis using The Cancer Genome Atlas (TCGA) database. We found that high levels of *CLOCK* were positively associated with advanced clinical stages and metastasis status of lung adenocarcinoma (LUAD) based data in UALCAN Network [32] (Fig. 6G-I). Furthermore, *CLOCK* expression was positively associated with *ALCAM*, *NANOG* and *POU5F1* expressions according to the data in GEPIA2 Network [33] (Fig. S5D-F). Consistently, the positive correlation between *ADRB2* and *CLOCK* expressions was confirmed in both lung squamous carcinoma (LUSC) and LUAD based the data in TIMER2 Network [34] (Fig. S5G, H). Importantly, lung cancer patients with elevated expression of CLOCK signatures exhibited significantly poor survival rates using Kaplan–Meier Plotter analysis [35, 36] (Fig. 6J–L). Altogether, these findings suggest that CLOCK is a potential biomarker and a therapeutic target for lung cancer patients under chronic stress.

## Discussion

Accumulating evidence reveals that chronic stress is associated with high mortality and poor prognosis in patients with cancer [37, 38]. Chemoresistance in lung cancer patients remains a major obstacle in clinical practice [16]. However, more effective therapeutic strategy for patients suffering from lung cancer and chronic stress is under urgent need. In this study, we demonstrated that the atypical antipsychotic agent OLZ suppressed chronic stress-enhanced lung tumorigenesis and anxiety-like behaviors in both *Kras*^*LSL−G12D/WT*^ lung cancer model and LLC1 syngeneic tumor model. OLZ inhibited stress-induced neuro-activity in mPFC and NE releasing to suppress the activation of ADRB2-cAMP-PKA-CREB pathway, which reduced phosphorylated CREB that transactivates CLOCK to enhance lung cancer stemness. As such, OLZ downregulated CLOCK expression to reverse chronic stress-enhanced chemoresistance to first-line anti-cancer agent GEM. Moreover, CLOCK expression was positively correlated with ADRB2 expression, serum NE, anxiety state and cancer development in patients with lung cancer, suggesting CLOCK expression as a promising prognostic factor for chronic stress-related lung cancer.

Increasing studies have provided potential therapeutic strategies for chronic stress-related cancer by exploring the mechanism by which chronic stress promotes cancer development. Chronic stress activates cAMP/PKA signaling pathway through adrenergic receptor ADRB2, leading to increased expression of VEGF, MMP2 and MMP9 to promote angiogenesis and tumor growth [12]. This study suggests ADRB blockage as a promising therapeutic strategy for ovarian cancer. In immunodeficient mouse model with breast cancer, chronic stress-induced epinephrine activates key enzyme LDHA to produce lactic acid, forming an acidic microenvironment to facilitate USP28-mediated deubiquitination of MYC, resulting in enhanced cancer stemness. Targeting LDHA with vitamin C reverses provides a strategy to ameliorate chronic stress-enhanced tumorigenesis [26]. Our study demonstrated that OLZ benefited cancer treatment not only physically but also psychologically. OLZ significantly reversed both chronic stress-induced tumorigenesis and anxiety-like behavior in both *Kras*^*LSL−G12D/WT*^ lung cancer model and LLC1 syngeneic tumor model. Furthermore, OLZ is applied in cancer chemotherapy as an antiemetic agent [19]. Thus, exploring the therapeutic role of OLZ in cancer treatment will benefit patients undergoing chemotherapy. A previous study revealed that OLZ enhances the anti-tumor activity of temozolomide in U87MG and A172 glioblastoma cell lines [20]. Also, a combination regime of OLZ and other GSK3β-inhibitors improves the prognosis of patients with refractory glioblastoma [39]. However, whether OLZ alone ameliorates chemoresistance in vivo remains unknown. Here, we found that chronic stress-induced NE promoted chemoresistance of lung cancer to GEM treatment, while OLZ increased the sensitivity of lung cancer to GEM as shown by suppressed proliferation and tumor growth.

The mechanism by which OLZ improves anti-cancer therapy is largely unexplored, as most previous studies focused on the effect of OLZ on tumor cells. OLZ is reported to enhance proliferation, migration and anchorage-independent growth of glioblastoma cells in vitro [20]. OLZ increases autophagic flux and autophagic vesicles by decreasing the translocation of p65 into the nucleus to prevent NF-κB activation, thus inducing autophagy in glioma cells [40]. In addition, OLZ sensitizes lung and pancreatic cancer stem cell lines to 5-fluorouracil, gemcitabine, and cisplatin by decreasing the expression of survivin, a protein involved in multidrug chemoresistance [41]. However, whether olanzapine exhibits an anti-cancer effect by modulating neuro-endocrine system remains to be further confirmed. As an antipsychotic drug, OLZ modulates neuro-activity in brain, for example, OLZ increases c-Fos immunoreactivity in the mPFC, nucleus accumbens shell (NAs), nucleus accumbens shell core (NAc) and dorsolateral striatum (DLSt), together with other brain regions responsible for incentive motivation and reward processing [25]. Our study demonstrated that OLZ decreased c-Fos immunoreactivity in the mPFC, leading to reduced NE-releasing. NE is a vital neurotransmitter underlying chronic stress-promoted tumor malignance [42]. We identified that NE upregulated circadian rhythm pathway leading to enhance cancer stem-like traits of NCI-H1299 lung cancer cell line with unbiased transcriptomic analysis. The core circadian regulator CLOCK/BMAL1 complex is reported to sustain cancer stem-like traits and immunosuppression features of glioblastoma [14]. Consistently, targeting the release of NE with OLZ suppressed CLOCK expression to reverse stress-enhanced lung cancer stemness. Yet, the mechanism by which NE regulates CLOCK rather than other core circadian genes to promote cancer stemness remains unexplored. NE abrogates the tumor-suppressing effect of anti-angiogenic agent sunitinib by activating β-adrenoceptor (ADRB)-cAMP-PKA signaling pathway to increase the expression of vascular endothelial growth factor (VEGF) and interleukin-8 (IL-8) [43]. Our study revealed that chronic stress-induced NE activated ADRB2-cAMP-PKA-CREB pathway to promote transcription of CLOCK. As CLOCK is a transcriptional factor, it is challenging to directly target the circadian core genes pharmacologic agents rather than targeting their regulators [44]. Clinical evidence identified a positive correlation between CLOCK expression and NE level, and CLOCK served as a promising indicator of chronic stress, cancer development and poor prognosis in patients with lung cancer.

## Conclusion

In this study, we showed that OLZ ameliorates chronic stress-enhanced anxiety and lung cancer stemness. OLZ suppressed the mPFC activity and the releasing of NE, which prevented NE-induced activation of ADRB2-cAMP-PKA-CREB pathway to promote oncogenic CLOCK expression via transactivation. Importantly, CLOCK-deficiency or OLZ alleviated chronic stress-enhanced chemoresistance to anti-cancer agent GEM.

## Electronic supplementary material

Below is the link to the electronic supplementary material.

Supplementary Material 1

Supplementary Material 2

## Data Availability

RNA-Seq data were deposited into the Gene Expression Omnibus database under accession number GSE268860.

## Abbreviations

mPFC: Medial prefrontal cortex
OLZ: Olanzapine
HADS: Hospital Anxiety and Depression Scale
NE: Norepinephrine
ADRB2: β2-adrenoceptor
GEM: Gemcitabine
BDNF: Brain-derived neurotrophic factor
ACTH: Adrenocorticotropic hormone
HPA: Hypothalamic-pituitary-adrenal
SNS: Sympathetic nervous system
VTA: Ventral tegmental area
TH: Tyrosine hydroxylase
ATP: Adenosine triphosphate
cAMP: Cyclic 3′-5′ adenosine monophosphate
PKA: Protein kinase A
CREB: cAMP response element binding protein
CBP: Carboplatin
CDDP: Cisplatin
GEM: Gemcitabine
PEM: Pemetrexed
PTX: Paclitaxel
IACUC: Institutional Animal Care and Use Committee
WT: Wild type
EPM: Elevated plus maze
OFT: Open field test
Cg1: Cingulate cortex
DEGs: Differentially expressed genes
PrL: Prelimbic cortex
IL: Infralimbic corex
DP: Dorsopeduncular cortex
NAs: Accumbens shell
NAc: Accumbens shell core
DLSt: Dorsolateral striatum
VEGF: Vascular endothelial growth factor
IL-8: Interleukin-8
LLC1: Lewis lung carcinoma
LUAD: Lung adenocarcinoma
LUSC: Lung squamous carcinoma
Micro-CT: Micro-computed tomography
ELDA: Extreme limiting dilution assay
ELISA: Enzyme-linked immunosorbent assay
