# Supplementary material for: Olanzapine suppresses mPFC activity-norepinephrine releasing to alleviate CLOCK-enhanced cancer stemness under chronic stress
Source: Cell Commun Signal. 2024 Jul 25;22:375. doi: 10.1186/s12964-024-01747-y (PMC11270788; doi:10.1186/s12964-024-01747-y)

Full unedited gel for Fig. 1C

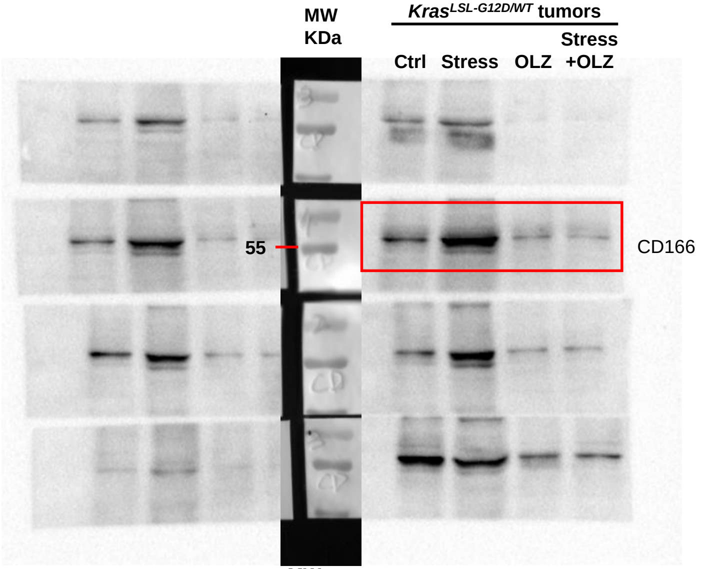

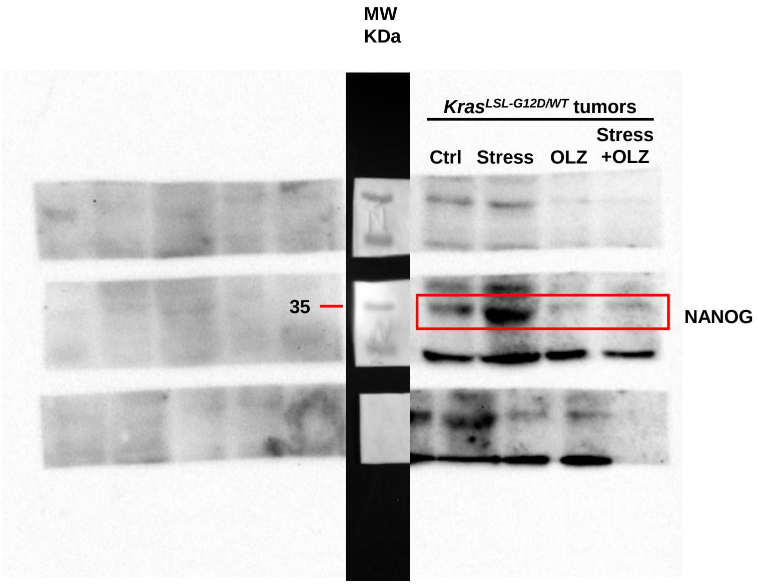

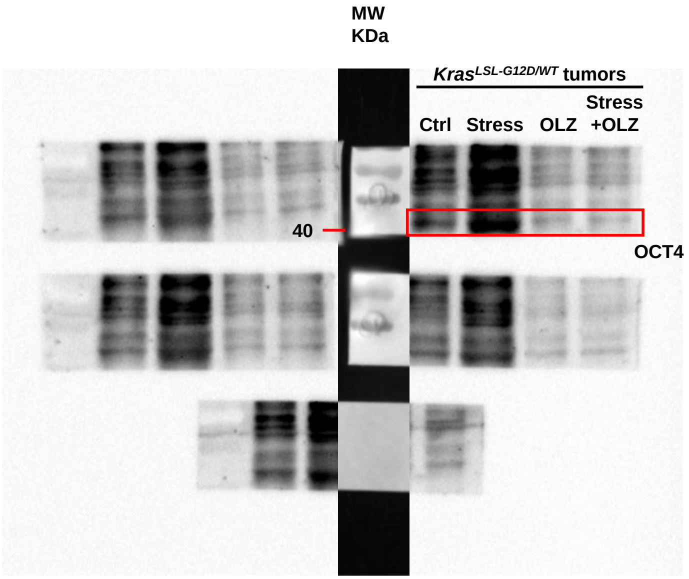

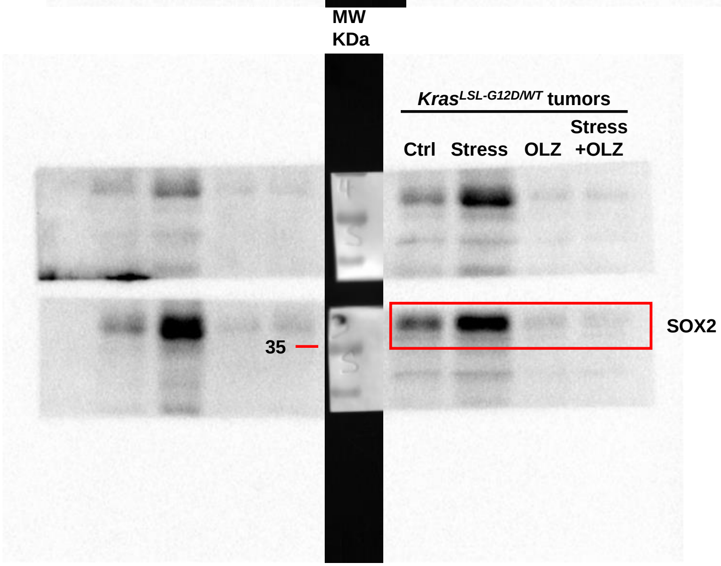

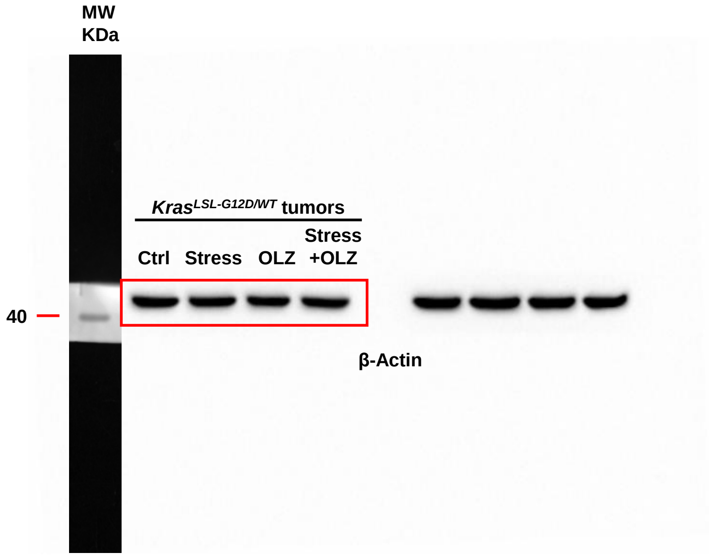

Full unedited gel for Fig. 3D

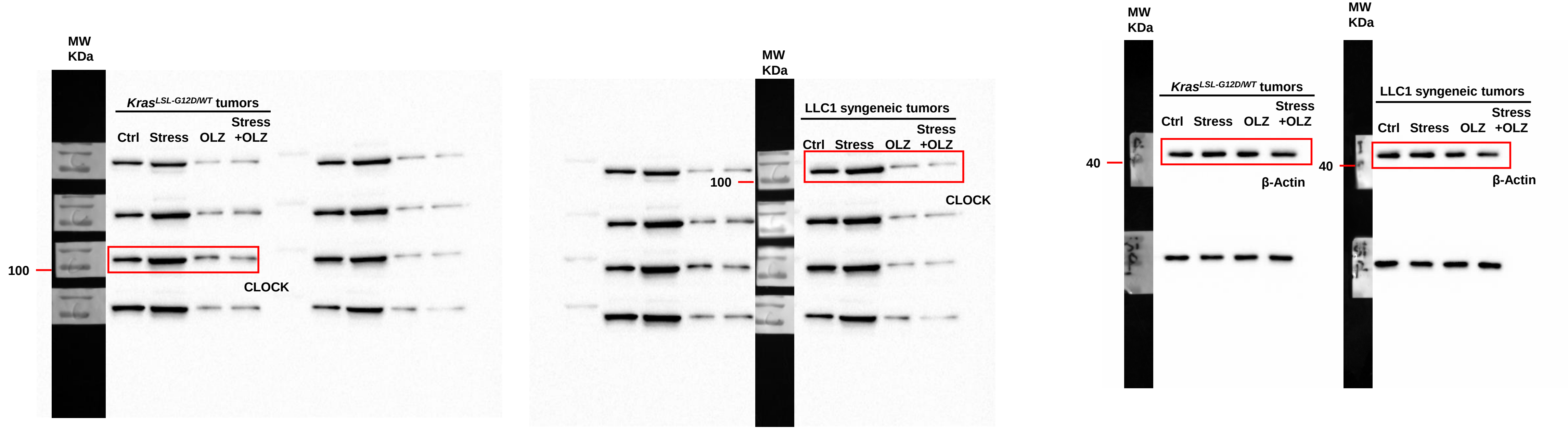

Full unedited gel for Fig. 3G

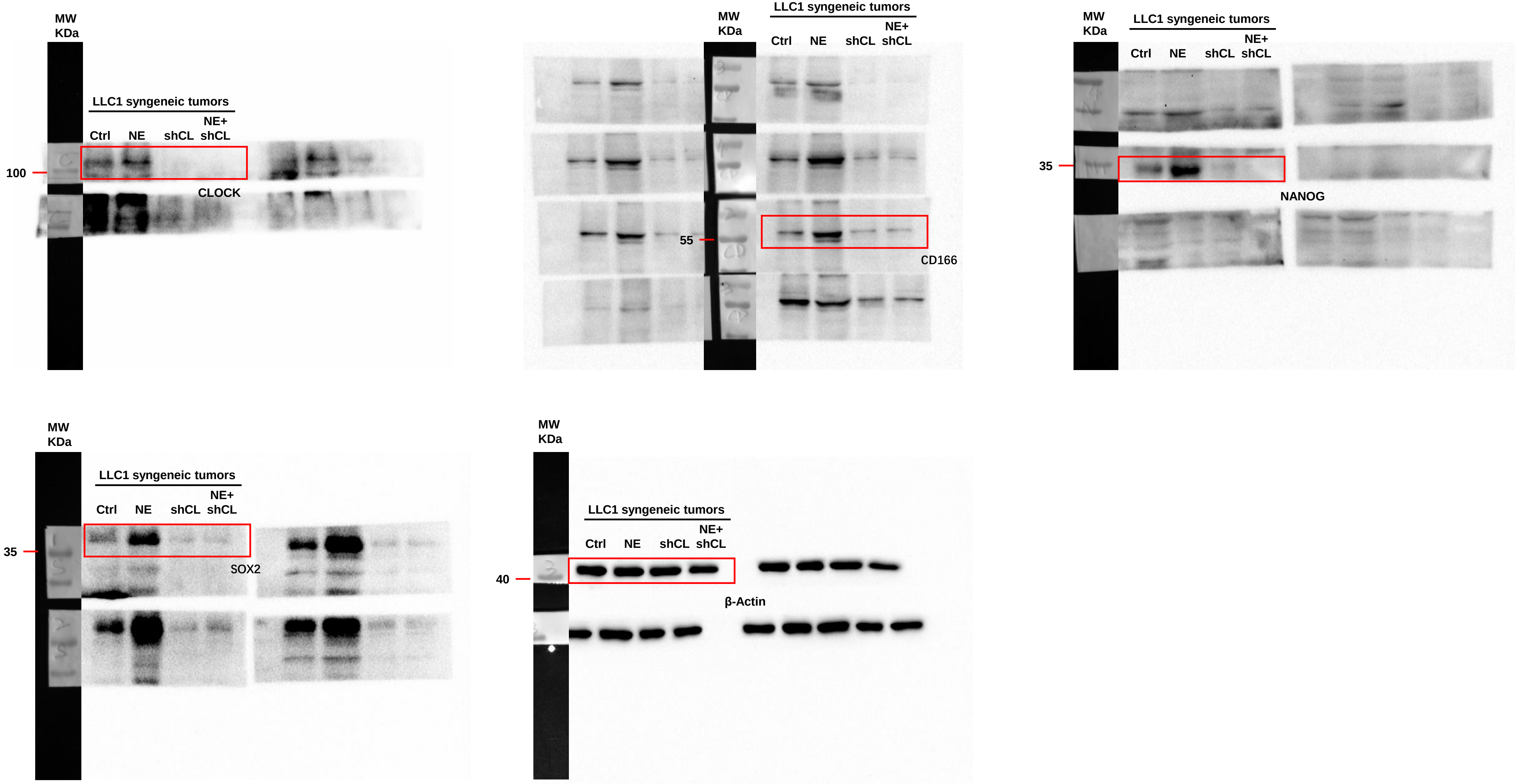

Full unedited gel for Fig. 4A

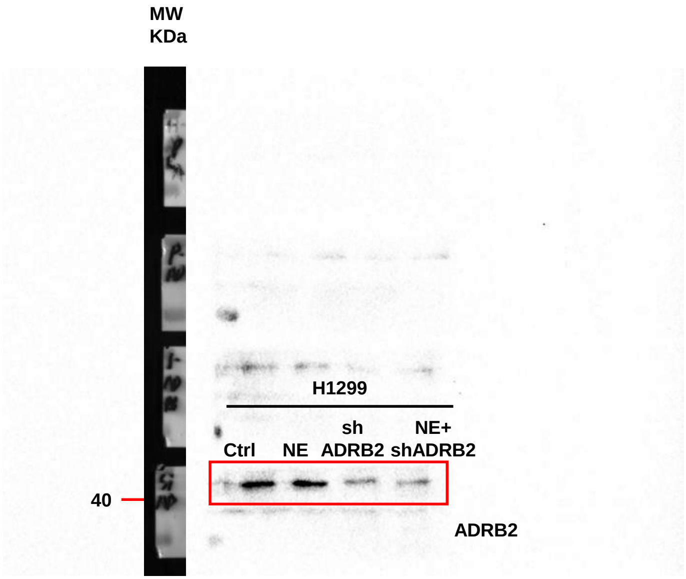

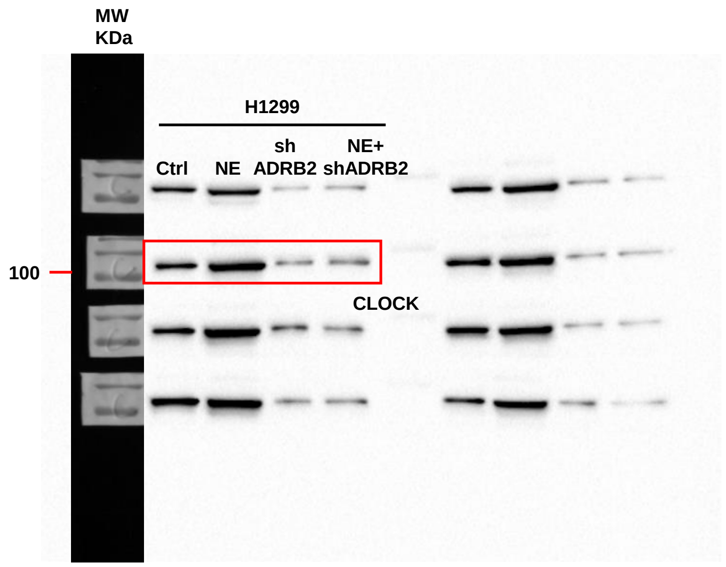

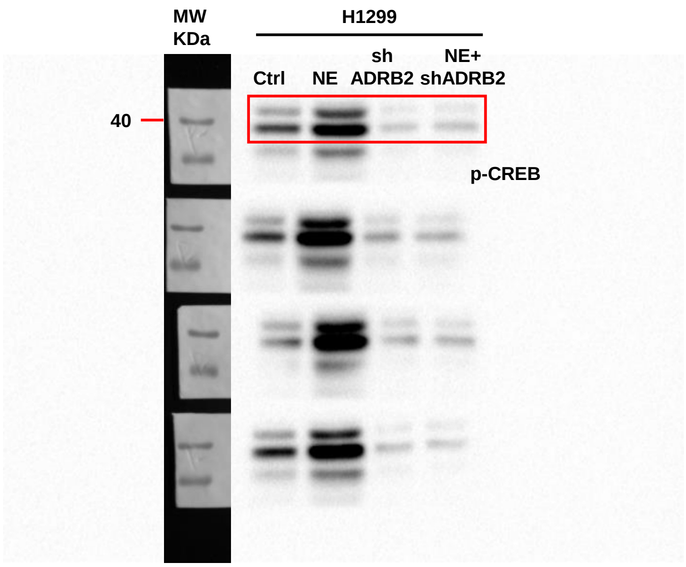

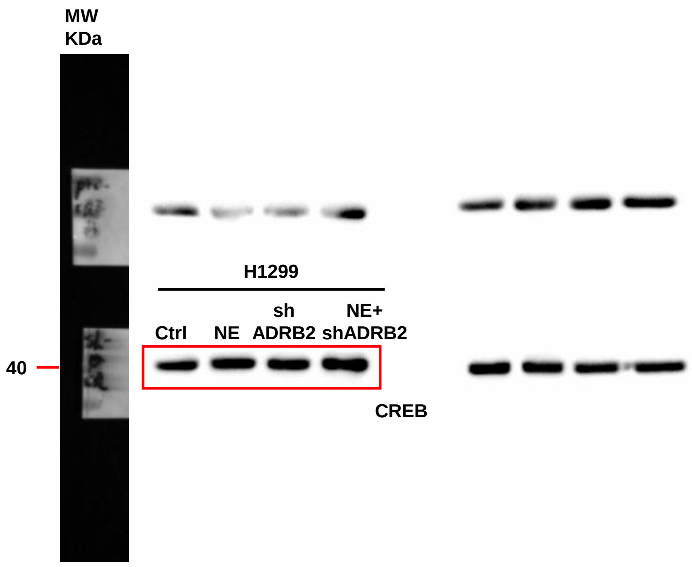

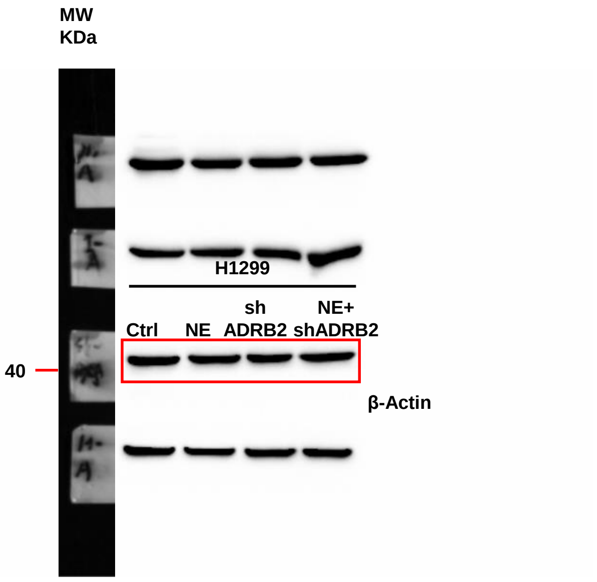

Full unedited gel for Fig. 4C

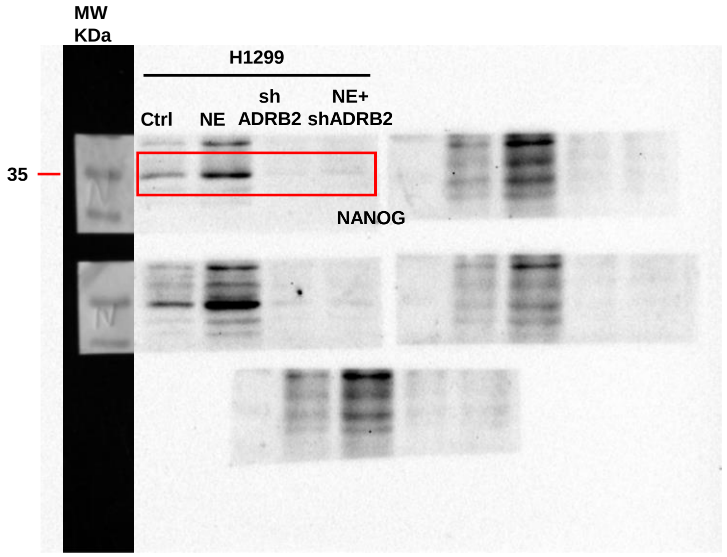

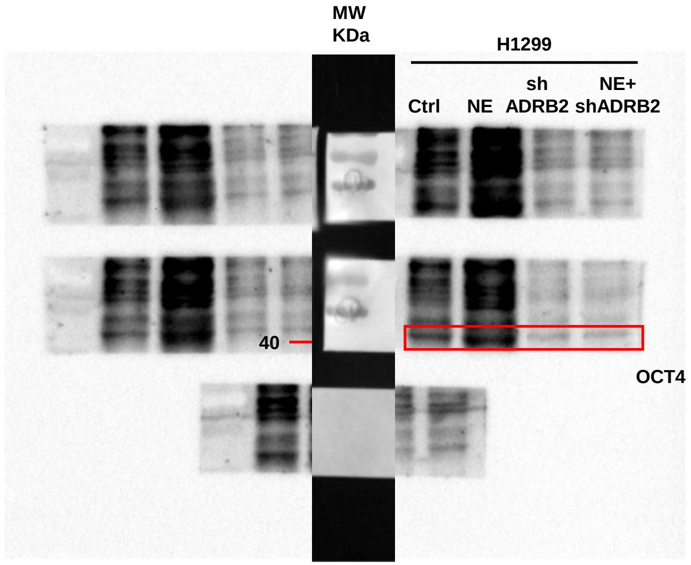

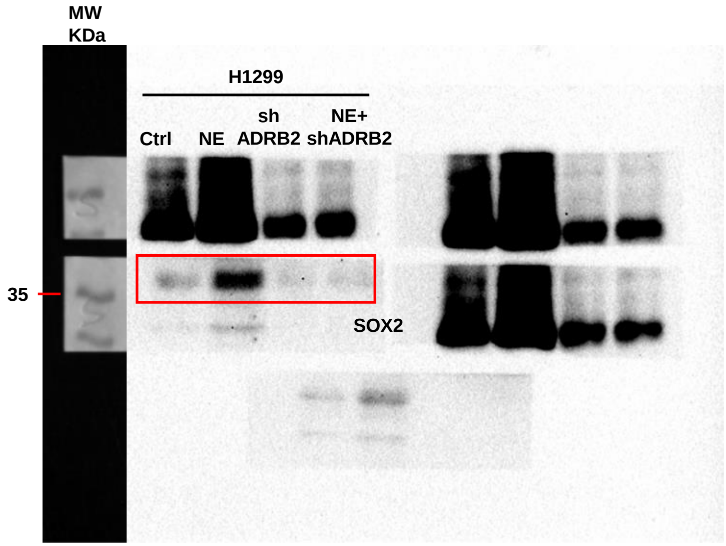

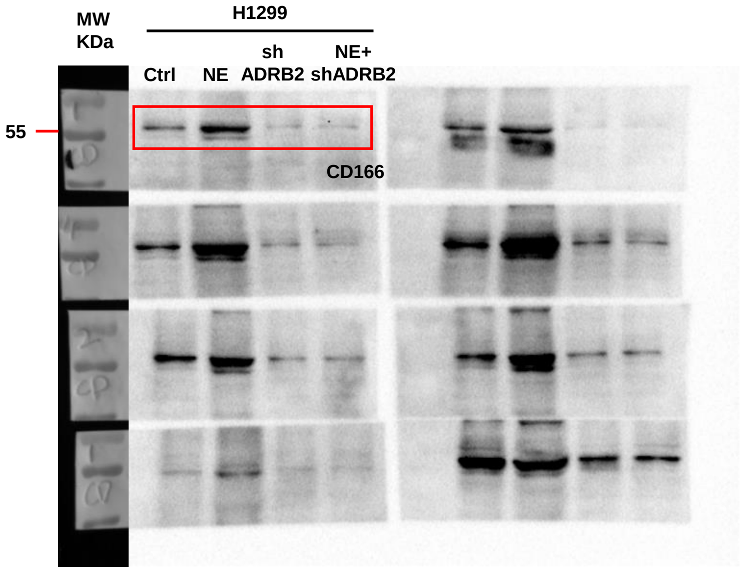

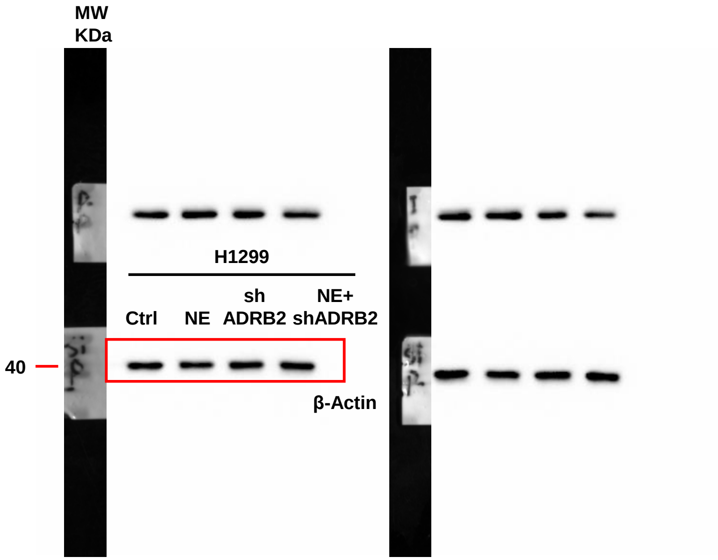

Full unedited gel for Fig. 4E

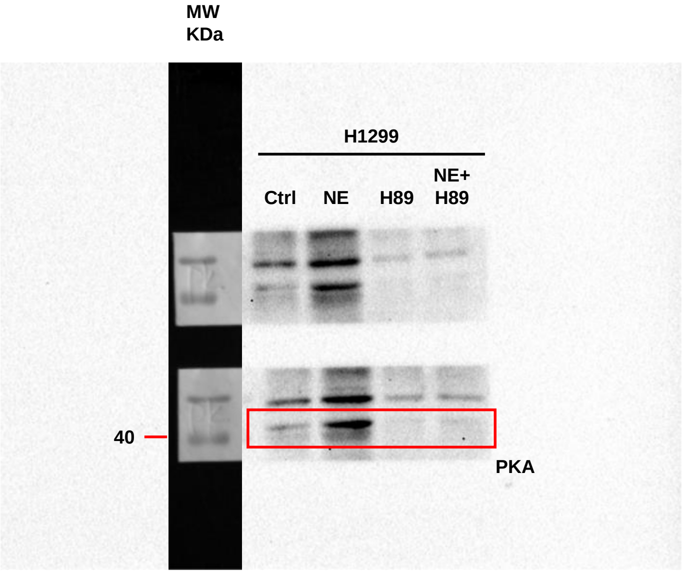

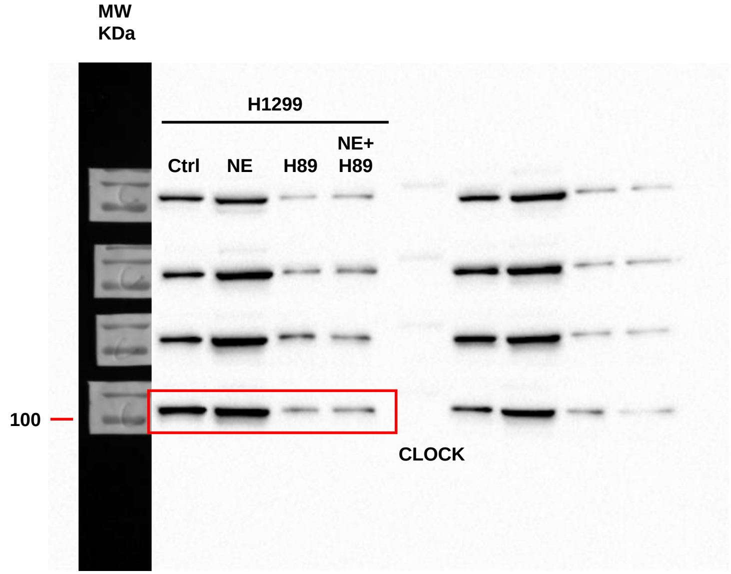

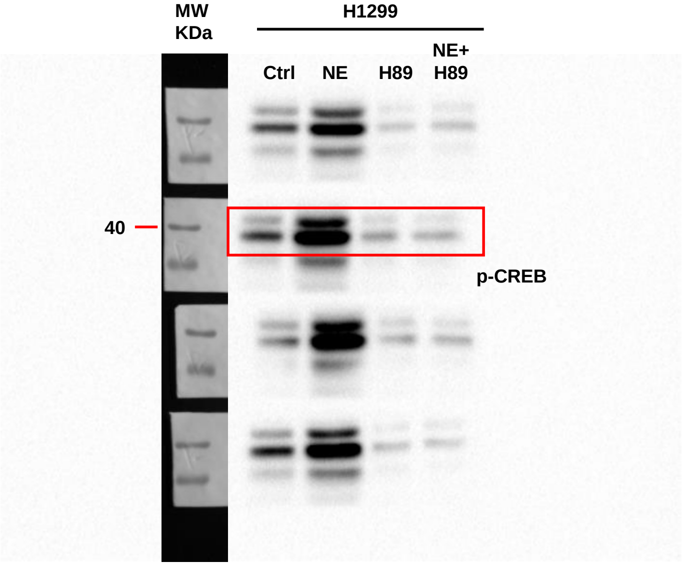

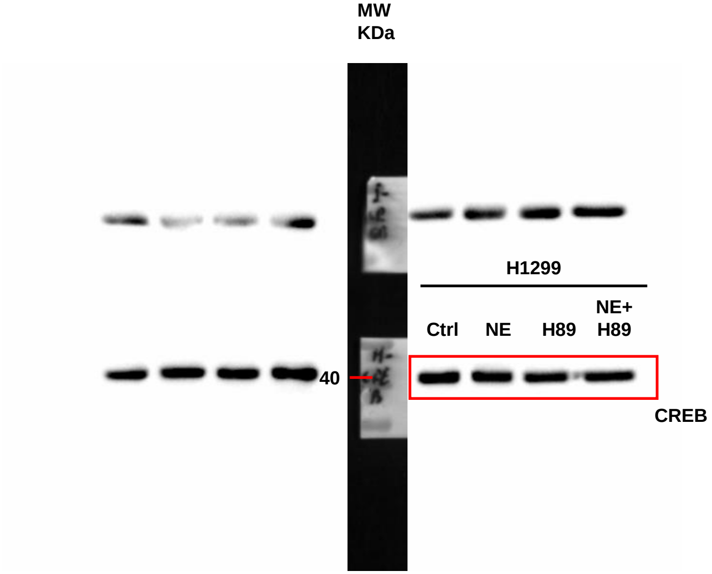

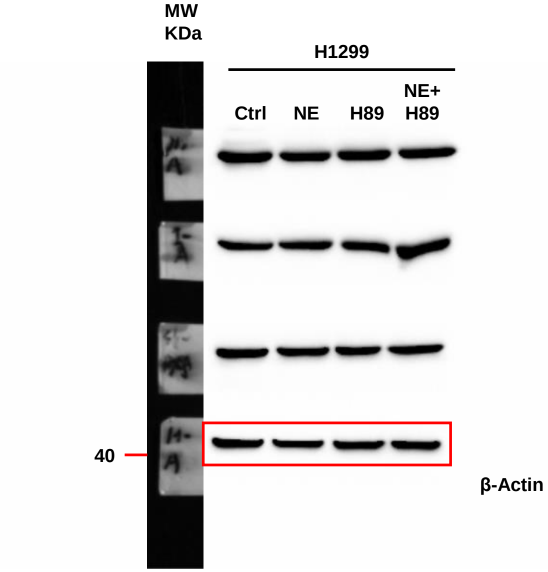

Full unedited gel for Fig. 4G

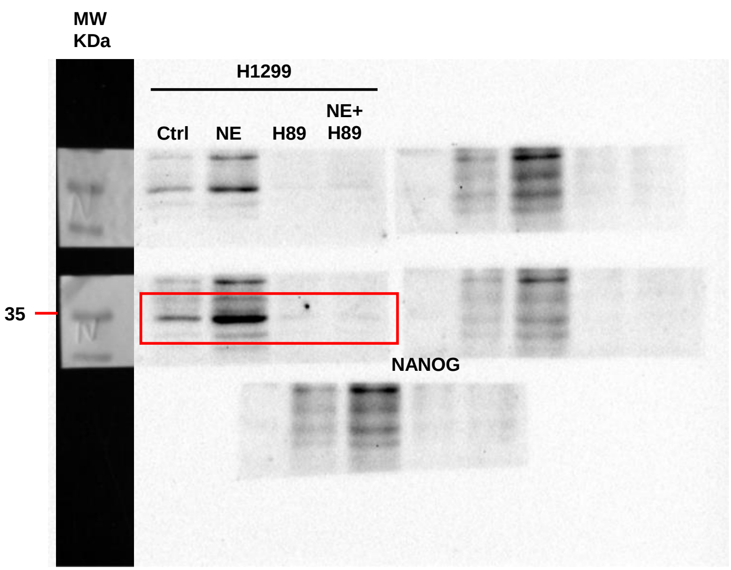

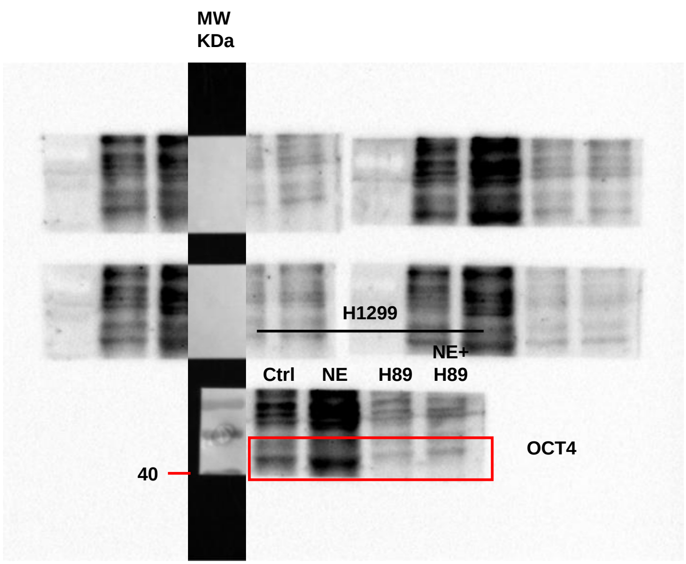

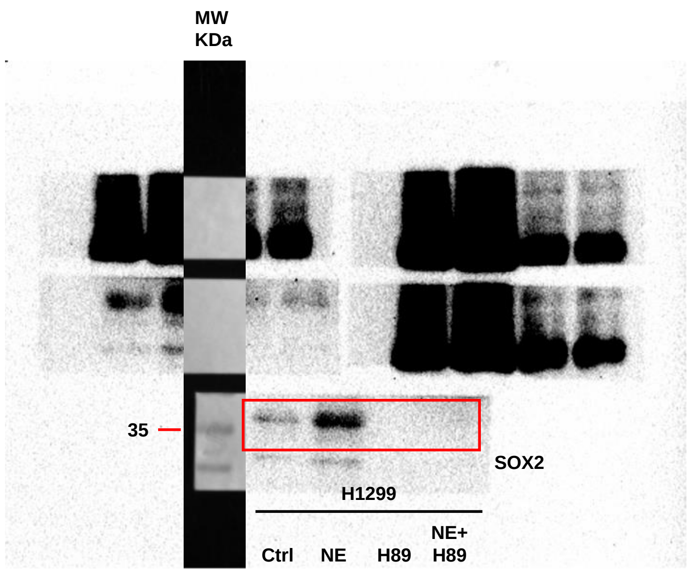

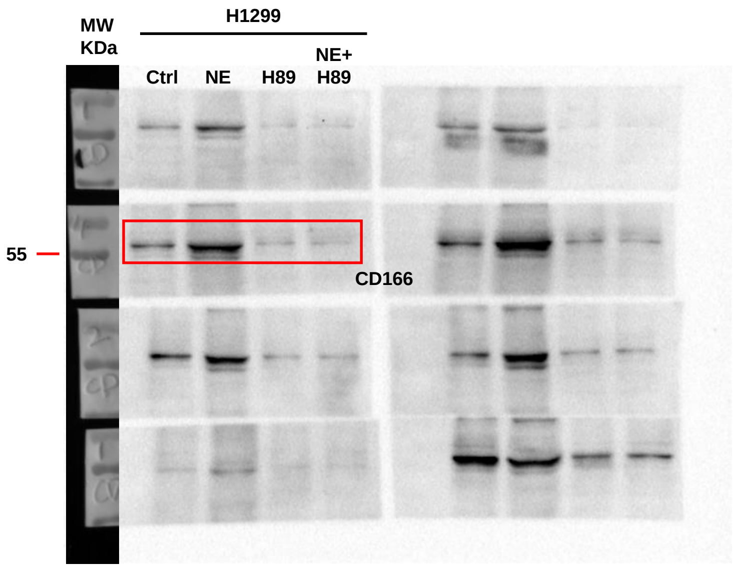

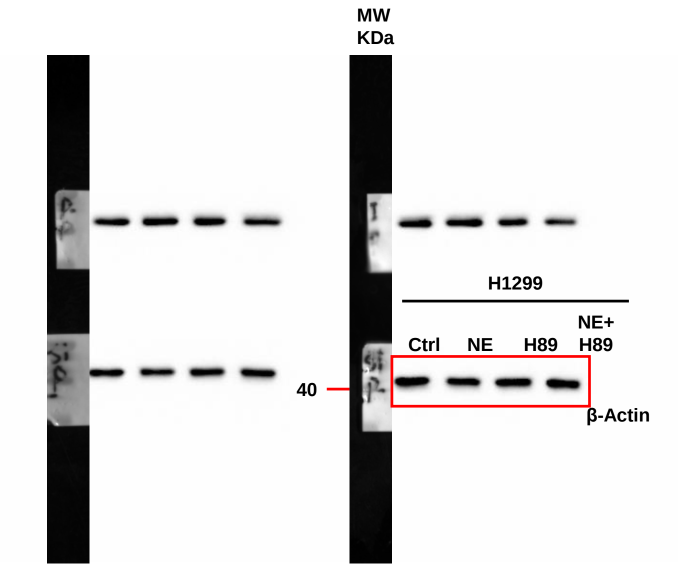

Full unedited gel for Fig. 6F

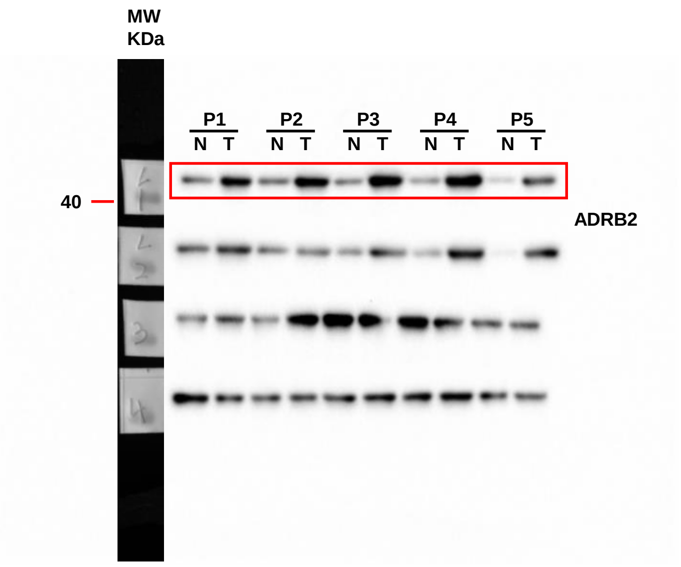

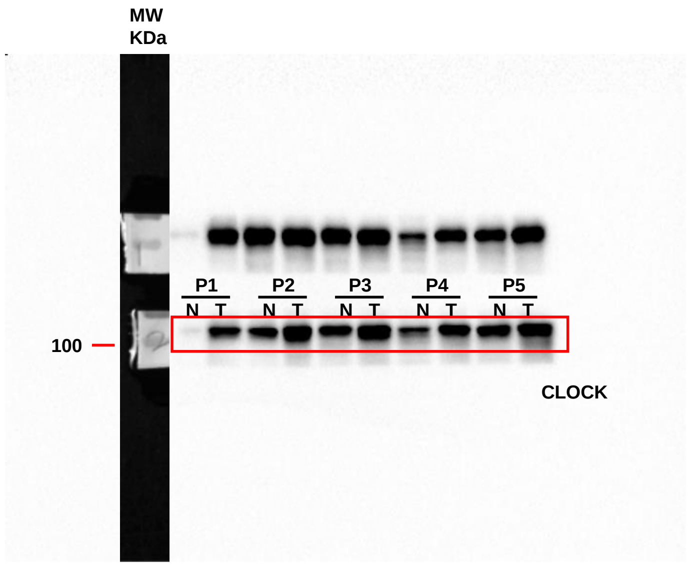

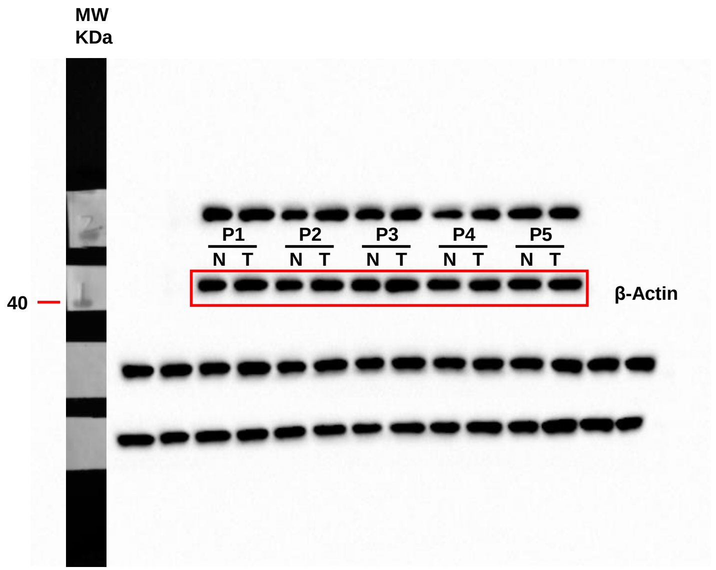

Full unedited gel for sFig. 1C

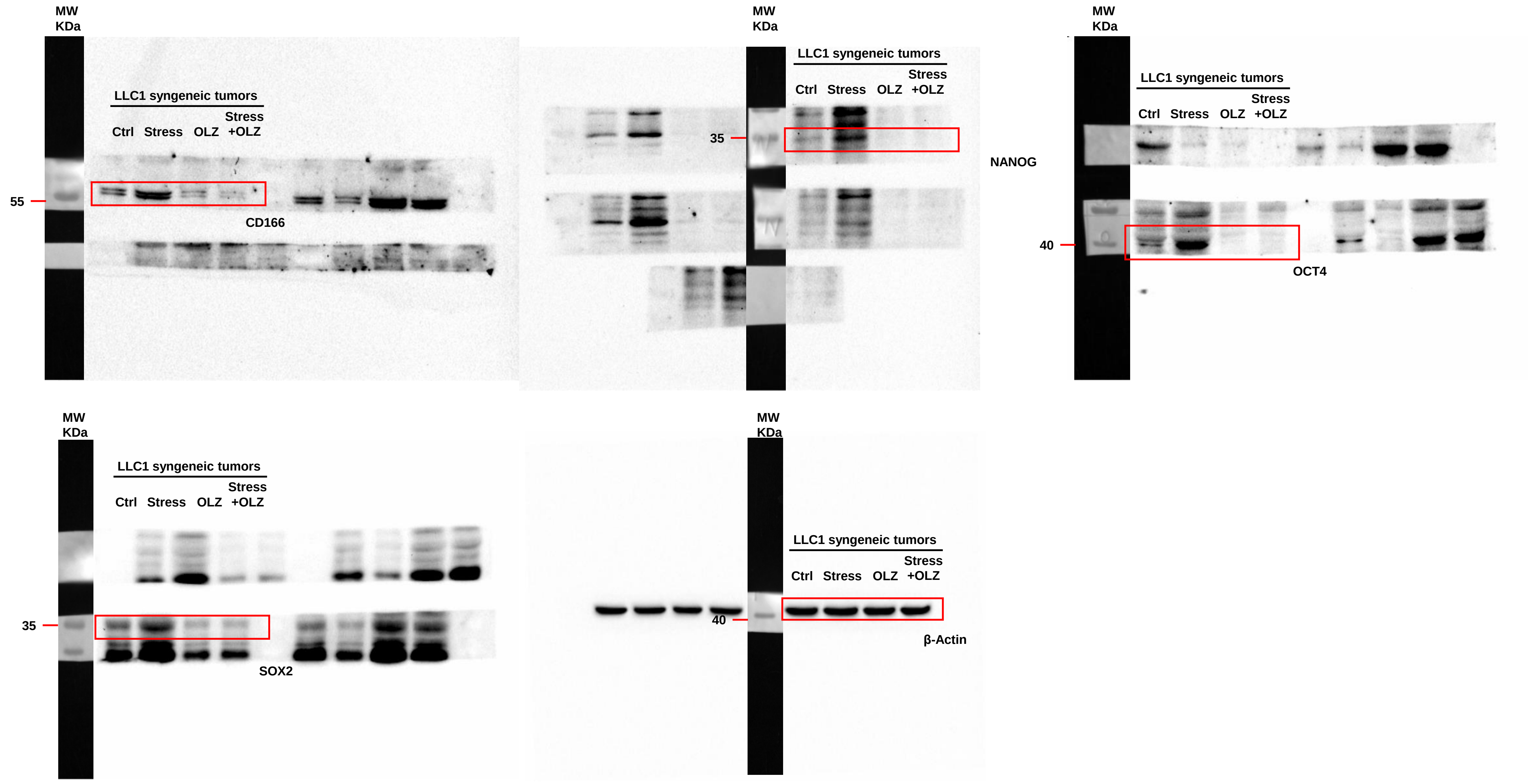

Full unedited gel for sFig. 3B

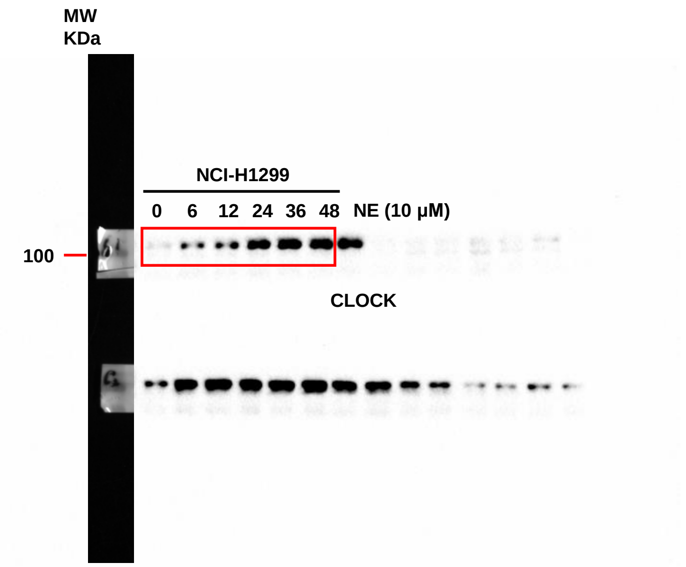

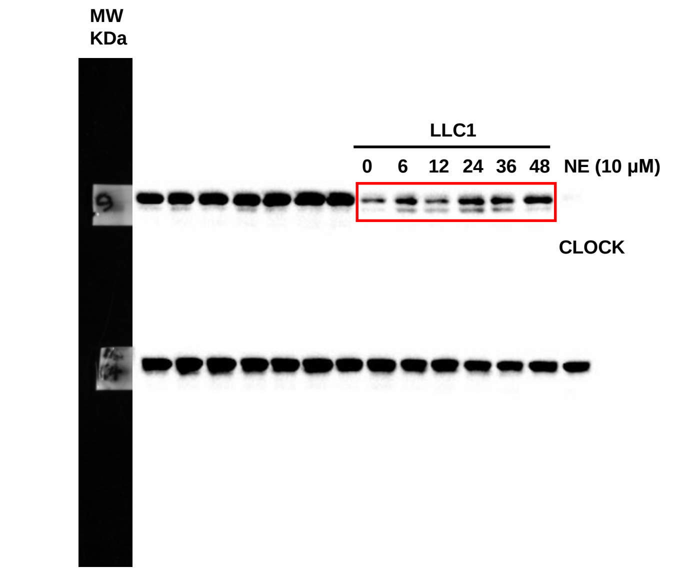

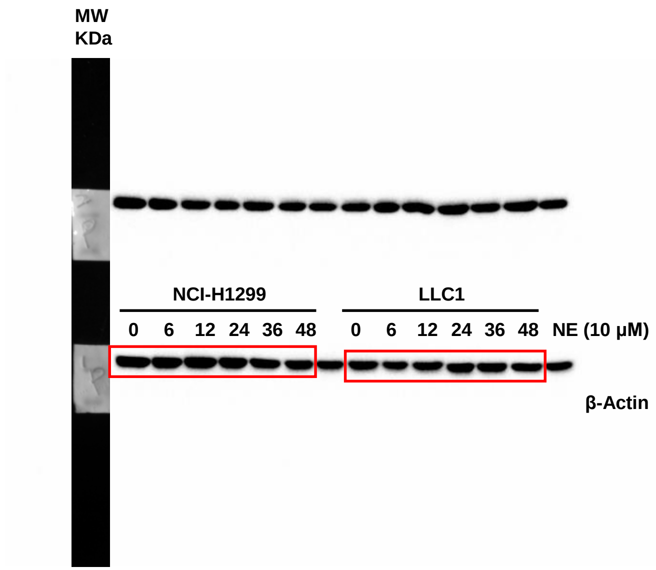

Supplement: Supplementary file 1 — Supplementary Material 1 [file 12964_2024_1747_MOESM1_ESM.pdf]
