# Supplementary material for: Olanzapine suppresses mPFC activity-norepinephrine releasing to alleviate CLOCK-enhanced cancer stemness under chronic stress
Source: Cell Commun Signal. 2024 Jul 25;22:375. doi: 10.1186/s12964-024-01747-y (PMC11270788; doi:10.1186/s12964-024-01747-y)
Supplement: Supplementary file 2 — Supplementary Material 2 [file 12964_2024_1747_MOESM2_ESM.pdf]

Supplemental Information for Manuscript "Olanzapine suppresses mPFC activity-norepinephrine releasing to alleviate CLOCK-enhanced cancer stemness under chronic stress"

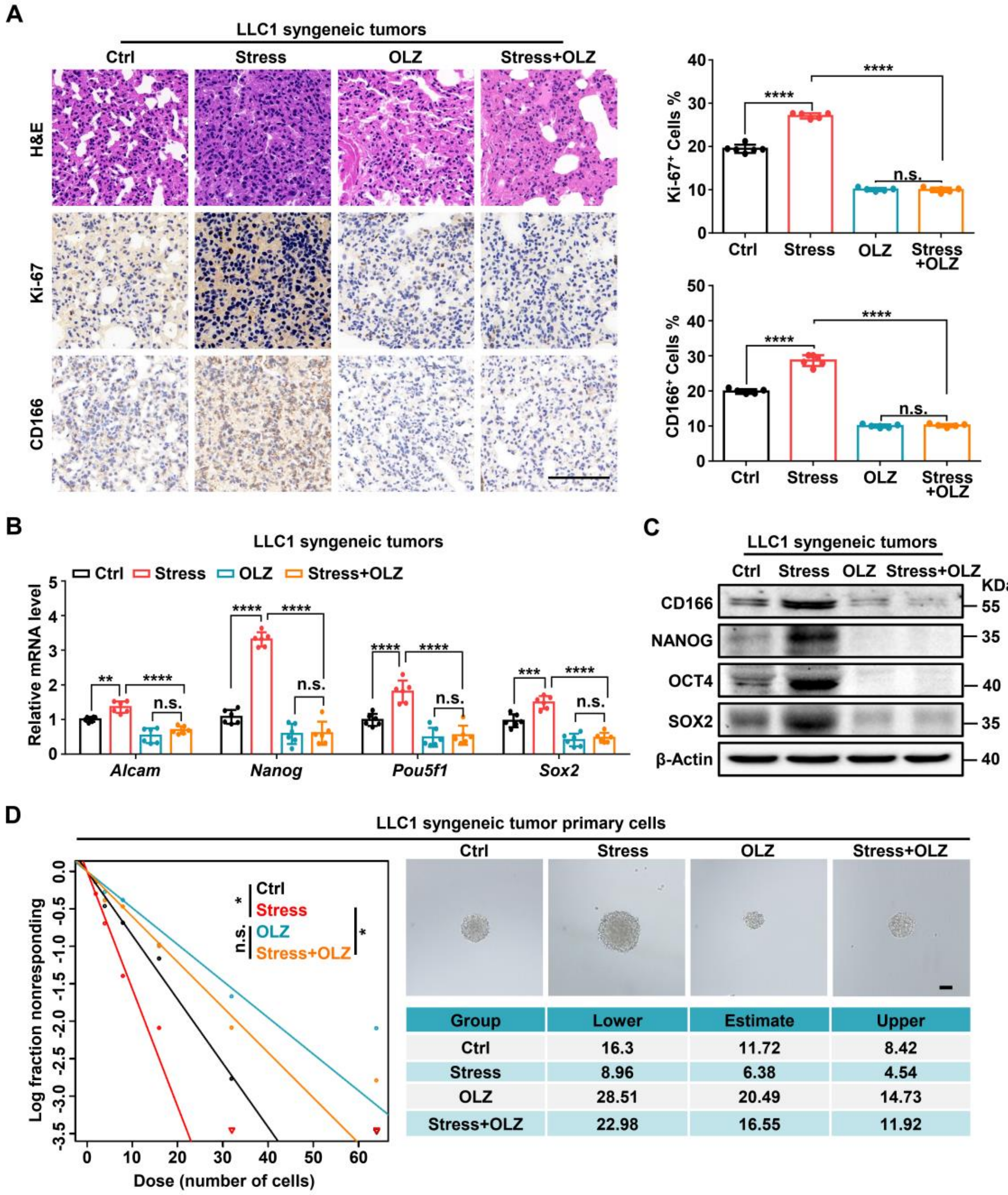

Fig. S1 OLZ suppresses chronic stress-enhanced lung tumorigenesis. (A) Representative typical H&E and IHC staining (left) and quantification (right) of the indicated markers in vehicle control (i.p., 0.1% DMSO in normal saline) or olanzapine (OLZ, i.p., 5 mg/kg/d) treated ctrl and stress of LLC1 syngeneic tumor in C57BL/6J mice. Scale bars, 100 μm. (B, C) Relative mRNA levels (B) and protein levels (C) of cancer stemness factors in LLC1 syngeneic tumor from (A). β-Actin was used as a

loading control. (D) Extreme limiting dilution analysis (ELDA) diagram (left), representative sphere images (top right), and stemness frequency (bottom right) of primary cells isolated from LLC1 syngeneic tumors in (A). Spheres were counted from 16 replicate wells. Scale bars, 100 μm.

Data in A and B represent the mean ± SEM (n = 6 mice each group). Statistical significances were determined using one-way ANOVA followed by Sidak's multiple comparison test (A, B) or likelihood ratio test (D) (* P < 0.05, ** P < 0.01, *** P < 0.001, **** P < 0.0001, n.s. P > 0.05).

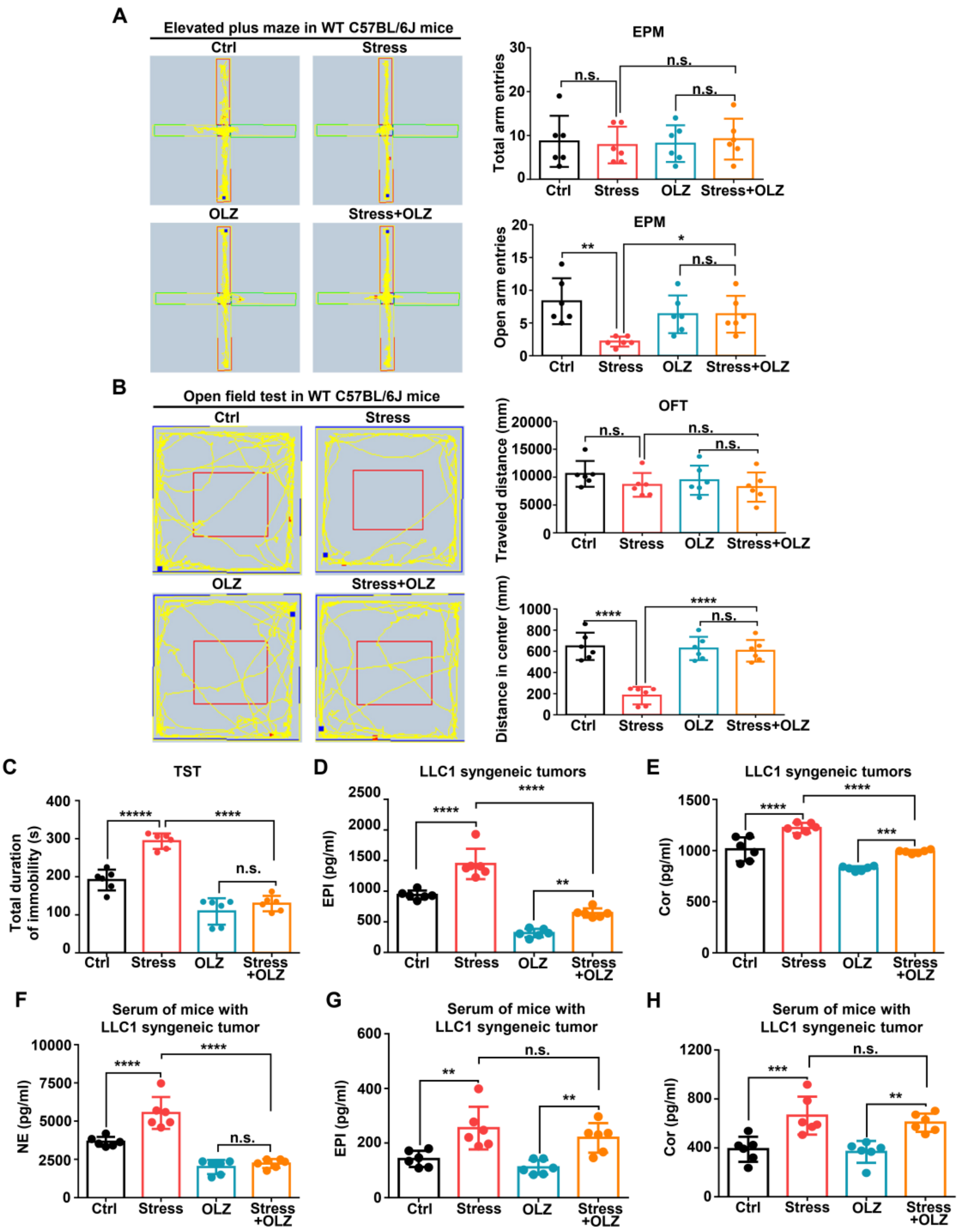

Fig. S2 OLZ relieves chronic stress-induced NE releasing and depression-like behavior in mice with lung cancer. (A) Representative typical image of elevated plus maze (EPM) (left) and quantification of total arm entries (top right) and open arm entries (bottom right) in C57BL/6J mice bearing with LLC1 syngeneic tumors treated with vehicle control (i.p., 0.1% DMSO in normal saline) or olanzapine (OLZ, i.p., 5

mg/kg/d) under Ctrl and stress. (B) Representative typical image of open field test (OFT) (left) and quantification of traveled distance (top right) and distance in center (bottom right) of C57BL/6J mice in (A). (C) Total duration of immobility in tail suspension test of C57BL/6J mice in (A). (D, E) Epinephrine (EPI) (D) and cortisol (Cor) (E) concentration of LLC1 tumors subcutaneously inoculated in C57BL/6J mice treated with vehicle control (i.p., 0.1% DMSO in normal saline) or OLZ (i.p., 5 mg/kg/d) under Ctrl and stress. (F-H) Serum level of NE (F), EPI (G) and Cor (H) in mice in (C).

All data represent the mean ± SEM (n = 6 mice each group). Statistical significances were determined using one-way ANOVA followed by Sidak's multiple comparison test (* $P < 0.05$, ** $P < 0.01$, *** $P < 0.001$, **** $P < 0.0001$, n.s. $P > 0.05$).

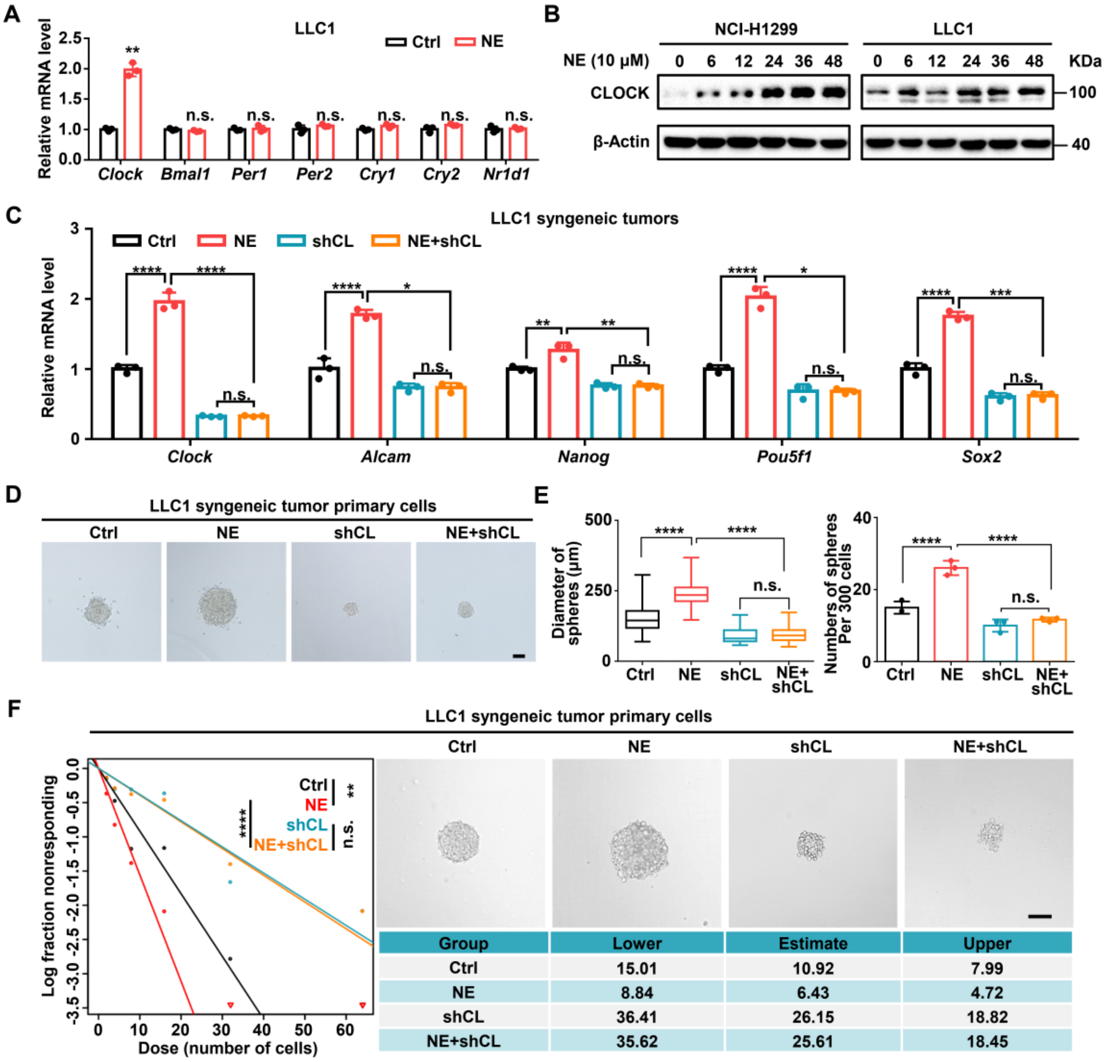

Fig. S3 Circadian gene CLOCK mediates NE-induced tumor growth and cancer stemness. (A) Relative mRNA levels of core circadian genes in LLC1 cells treated with NE (NE 10 μM, 48 h). (B) Relative protein levels of CLOCK in both NCI-H1299 and LLC1 cells treated with NE for indicated time. β-Actin was used as a loading control. (C) Relative mRNA levels of *Clock* and cancer stemness factors in the LLC1 syngeneic tumors with CLOCK silencing (shCL) and supplement of NE (s.c., 3 mg/kg/d). (D, E) Sphere formation ability was analyzed with the primary cells from LLC1 syngeneic tumors in (C). (D)The representative images were presented and (E) diameter (left) and the number (right) of spheroids were measured and counted. Scale bar, 100 μm. (F)

Extreme limiting dilution analysis (ELDA) diagram (left), representative sphere images (top right), and stemness frequency (bottom right) of primary cells isolated from LLC1 syngeneic tumors in (C) Spheres were counted from 16 replicate wells. Scale bars, 100 μm.

All data represent the mean ± SEM (n = 3 independent experiments). Statistical significances were determined using two tailed unpaired Student's t test (A), one-way ANOVA followed by Sidak's multiple comparison test (C, E) or likelihood ratio test (F) (* $P < 0.05$, ** $P < 0.01$, *** $P < 0.001$, **** $P < 0.0001$, n.s. $P > 0.05$).

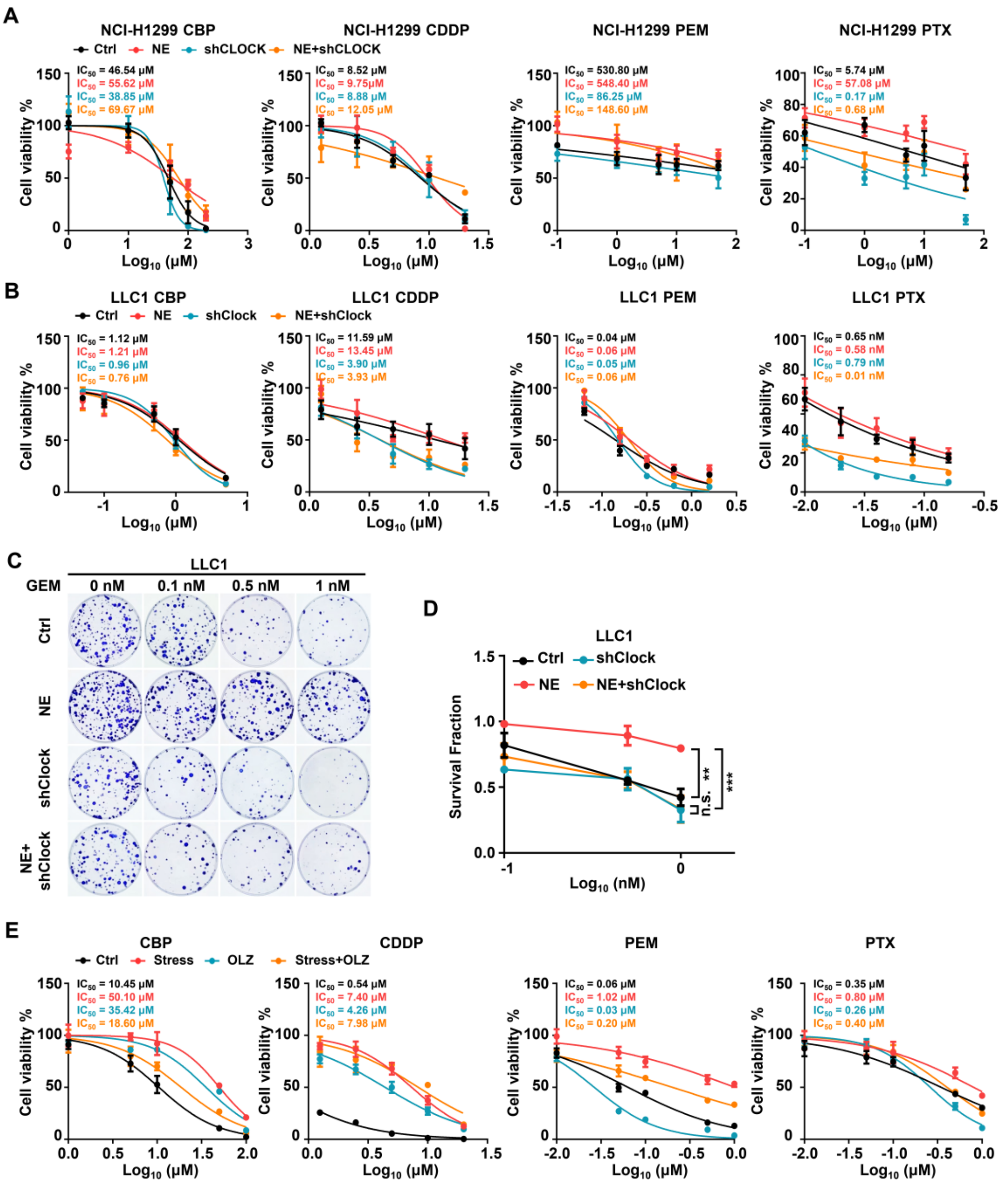

Fig. S4 Ablation of CLOCK or OLZ restrains chronic stress-/NE-induced chemotherapy resistance in lung cancer. (A) NCI-H1299 cells followed depletion of CLOCK (shCLOCK) and supplement of NE then treated with different concentrations of carboplatin (CBP), cisplatin (CDDP), pemetrexed (PEM) or paclitaxel (PTX) for 48 h. Cell viability was determined by CCK-8 assay. The numbers in the figure keys represent the concentrations (μM) of CBP, CDDP, PEM or PTX. Cells treated with

vehicle serve as a blank control. (B) Cell viability of LLC1 cells followed depletion of CLOCK (shClock) and supplement of NE then treated with different concentrations (μM) of CBP, CDDP, PEM or PTX. (C, D) Representative images (C) and quantification (D) of LLC1 colonies followed depletion of CLOCK (shClock) and supplement of NE then treated with 0, 0.1, 0.5 and 1 nM gemcitabine GEM. (E) Cell viability of primary LLC1 cells in the determined groups treated with different concentrations (μM) of CBP, CDDP, PEM or PTX.

Data in A, B, E represent the mean ± SEM (n = 5 independent experiments). Data in D represent the mean ± SEM (n = 3 independent experiments). Statistical significance was determined using one-way ANOVA followed by Sidak's multiple comparison test (D) (** $P < 0.01$, *** $P < 0.001$, n.s. $P > 0.05$).

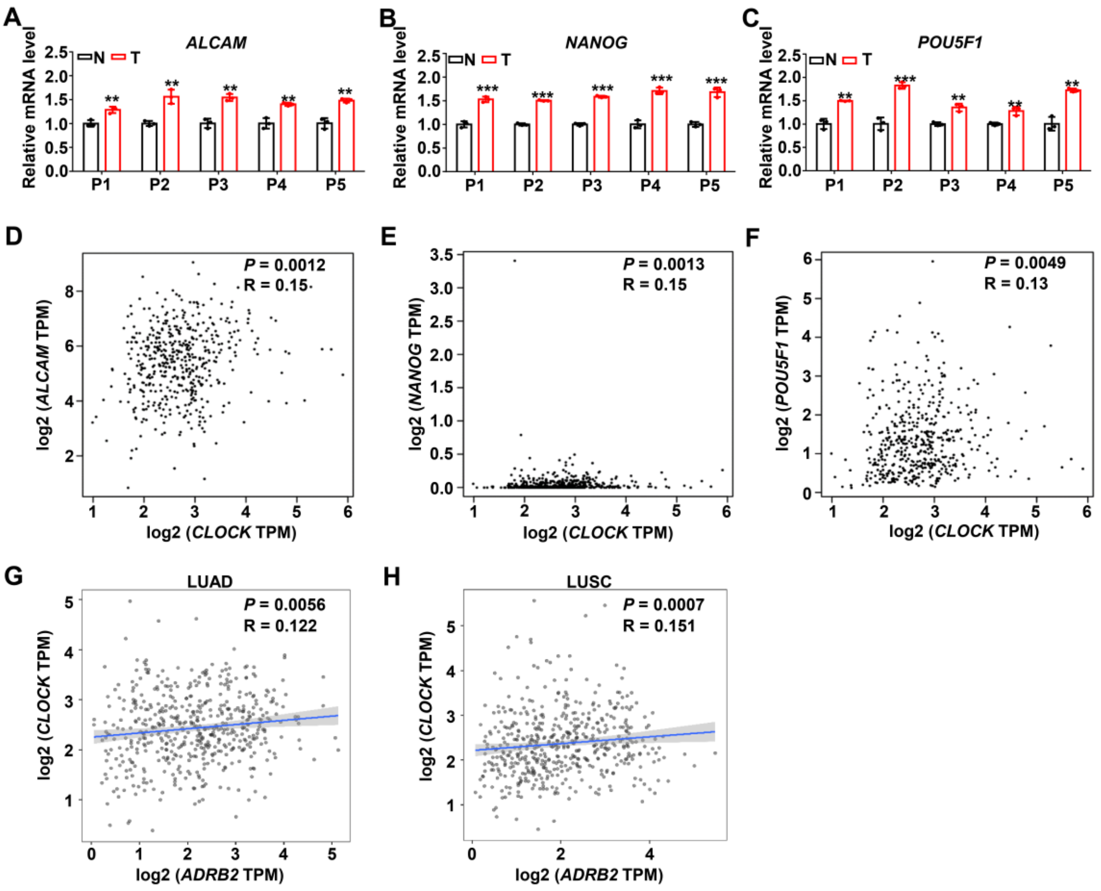

Fig. S5 Clinical relevance of NE and CLOCK in patients with lung cancer CLOCK expression is positively associated with depression status, serum NE level and poor prognosis in lung cancer patients. (A, B, C) Relative mRNA levels of *ALCAM* (A), *NANOG* (B) and *POU5F1* (C) in adjacent normal tissues (N) and tumor tissues (T) in patients with lung cancer. (D, E, F) Linear regression analysis of the correlations between *CLOCK* and *ALCAM* (D), *NANOG* (E), and *POU5F1* (F) based upon data generated by the GEPIA2 Network. (G, H) Linear regression analysis of the correlations between *CLOCK* and *ADRB2* in lung adenocarcinoma (LUAD) (G) and lung squamous carcinoma (LUSC) (H) and based upon data generated by the TIMER2 Network.

Data in A, B, C represent the mean ± SEM (n = 3 independent experiments). Statistical significances were determined using two tailed unpaired Student's t test (A-H) or Pearson (R) correlation (D-H) (** $P < 0.01$, *** $P < 0.001$).

Table S1 Sequences of shRNAs

	Forward (5'-3')	Reverse (5'-3')
Hum_ shCLOCK	CCGGGCGAGGAACAATAGACCC AAACTCGAGTTTGGGTCTATTGT TCCTCGCTTTTT	AATTAAAAAGCGAGGAACAATAG ACCCAAACTCGAGTTTGGGTCTA TTGTTCCTCGC
Mus_ shClock	CCGGCTTCAGCAGTCAGTCCATA AACTCGAGTTTATGGACTGACTG CTGAAGTTTTT	AATTAAAAACTTCAGCAGTCAGT CCATAAACTCGAGTTTATGGACTG ACTGCTGAAG

Table S2 Sequences of cloning primers

Luciferase constructs	Forward (5'-3')	Reverse (5'-3')
pGL3-CLOCK-(-654, +1137)	GGGGTACCCCCGACCTCCTTTC GCTTCC	CCAAGCTTCGCCAGAGCCAAC TCCAGAA
pGL3-CLOCK-(-146, +1137)	GGGGTACCTCTCTTCGGGCGTC CGGGAT	CCAAGCTTCGCCAGAGCCAAC TCCAGAA
pGL3-CLOCK-(+497, +1137)	GGGGTACCTGGGGAAGGCGGG AGCTGAA	CCAAGCTTCGCCAGAGCCAAC TCCAGAA

Table S3 Sequences of primers for RT-qPCR

GENE	Forward (5'-3')	Reverse (5'-3')
ACTB	TTGCCGACAGGATGCAGAAGGA	AGGTGGACAGCGAGGCCAGGAT
MYC	GTCAAGAGGCGAACACACAAC	TTGGACGGACAGGATGTATGC
ALCAM	TCCTGCCGTCTGCTCTTCT	TTCTGAGGTACGTCAAGTCGG
POU5F1	GGGAGATTGATAACTGGTGTGTT	GTGTATATCCCAGGGTGATCCTC
SOX2	TGGACAGTTACGCGCACAT	CGAGTAGGACATGCTGTAGGT
NANOG	ACCTATGCCTGTGATTTGTGG	AGTGGGTTGTTTGCCTTTGG
CLOCK	CCTGAGACAGCTGCTGACAA	TAAATGCTGCCTGGGTGGAG
BMAL1	CCACAAAGATGGGGCTGGAT	CACCCTGATTTCCCCGTTCA
PER1	ACTCCTGCGACCAGGTACTGGCTG	GGCCACCACGGATGCACGA
PER2	GGATGCCCGCCAGAGTCCAGAT	TGTCCACTTTCGAAGACTGGTCGC
CRY1	CAGGTGGCGATTTTTGCTTCA	TCCAGCTTCAGTTGCCAGTT
CRY2	TGGAGCAGTCTGGACAGTCA	GAACCACGGGTCGAGAATGT
NR1D1	TGGACTCCAACAACAACACAG	GATGGTGGGAAGTAGGTGGG
ADRB2	TTGCTGGCACCCAATAGAAGC	CAGACGCTCGAACTTGGCA
Actb	GAGGTATCCTGACCCTGAAGTA	CACACGCAGCTCATTGTAGA
Alcam	ATGGCATCTAAGGTGTCCCCT	GTTGACAGTGTACCATCCAAGG
Myc	CCCTATTTCATCTGCGACGAG	GAGAAGGACGTAGCGACCG

Sox2	GCGGAGTGGAAACTTTTGTCC	CGGGAAGCGTGTACTTATCCTT
Nanog	CACAGTTTGCCTAGTTCTGAGG	GCAAGAATAGTTCTCGGGATGAA
Clock	CATCTAGGTTGCTCCACGGG	GGACCTTGGAAGGGTCAGTC
Bmal1	TGACCCTCATGGAAGGTTAGAA	GGACATTGCATTGCATGTTGG
Per1	CGGATTGTCTATATTTCGGAGCA	TGGGCAGTCGAGATGGTGTA
Per2	GGACTTCTCCCCAAGTCCCA	TGGAGCAGTTCTCGTTTCCG
Cry1	CACTGGTTCCGAAAGGGACTC	CTGAAGCAAAAATCGCCACCT
Cry2	TGCATCATTGGCGTGGACTA	GCTCCCAGCTTGGCTTGAAC
Nr1d1	GACGACCCTGGACTCCAATAA	TGAGGGAGCCAGTAGGTGAT
CLOCK -ChIP-1	GAGAGAGCTTTCGCTGGGGAC	AACGTGCCCGAGTGGTGA
CLOCK -ChIP-2	GTTTGGAGCAGCGCCCAATG	CACTAACGGAACCAGCCCG
CLOCK -ChIP-3	CGGGCTGGTTCCGTTAGTG	CGGGTGGACGGAAGAAGAGA
